# Sleep dysregulation in ADHD children: a systematic review and meta-analysis

**DOI:** 10.1017/S0033291725102158

**Published:** 2025-10-28

**Authors:** Peihua Xian, Xingxing Sheng, Sijia Liu, Zhiyuan Liu, Xiuyan Guo

**Affiliations:** 1 Shaanxi Key Laboratory of Behavior and Cognitive Neuroscience, School of Psychology, Shaanxi Normal University, Xi’an, China; 2 Fudan Institute on Ageing, Fudan University, Shanghai, China; 3 MOE Laboratory for National Development and Intelligent Governance, Fudan University, Shanghai, China

**Keywords:** attention-deficit/hyperactivity disorder (ADHD), children, sleep, meta-analysis

## Abstract

**Background:**

Attention Deficit Hyperactivity Disorder (ADHD) is a prevalent neurodevelopmental disorder in children. Abnormalities in sleep metrics among ADHD children gradually garnered attention. However, whether significant differences existed in sleep metrics between ADHD children and their typically developing (TD) counterparts remained controversial, with inconsistent conclusions across studies. Furthermore, the potential moderating effects of age and gender on these differential patterns remained insufficiently characterized.

**Methods:**

The current study systematically analyzed multimodal sleep monitoring data (polysomnography, actigraphy, electroencephalography, and questionnaires) from 34 articles spanning three decades (44 independent studies: 2,239 ADHD children vs. 57,181 TD children), focusing on core sleep metrics (total sleep time, sleep efficiency, sleep latency, wake after sleep onset, awakening index, and stage shifts) and their complex moderating mechanisms.

**Results:**

The results demonstrated that ADHD children exhibited impaired sleep continuity (reduced total sleep time, increased stage shifts), severe sleep interruption (prolonged wake after sleep onset, elevated awakening index), and abnormal sleep process effectiveness (decreased sleep efficiency, extended sleep latency). Demographic analyses revealed that maturation exacerbated ADHD-related sleep deficits, and male ADHD children had more severe sleep problems than female ADHD children. Furthermore, the moderating effect of gender composition on the awakening index showed interaction effects with other sleep metrics. In addition, slow-wave sleep acted as both a moderator and mediator in group differences of the awakening index.

**Conclusions:**

These findings provided novel neurodevelopmental explanations for sleep dysregulation in ADHD and proposed clinically translatable strategies involving gender-specific interventions and neuromodulation targeting slow-wave sleep.

## Introduction

Attention deficit hyperactivity disorder (ADHD) is a prevalent neurodevelopmental disorder characterized by symptoms such as inattention, hyperactivity, impulsivity, and difficulties in emotional regulation. Epidemiological studies have indicated that the prevalence of ADHD is 2.58% (Song et al., 2021), and the incidence rate among children reaches 6%–7% (Baji, Túri, Nagy, & Sterczer, 2024). This high prevalence rate has a significant impact on children’s daily functioning, academic performance, and social skills. Therefore, exploring intervention pathways for ADHD has become an important social issue of global concern.

Over the past few years, researchers have increasingly focused on the sleep problems of ADHD children, seeking to uncover their sleep characteristics as a strategy to identify new intervention approaches. Studies have shown that ADHD children exhibit significant sleep deficits or abnormalities (Craig, Weiss, Hudec, & Gibbins, 2020). Previous meta-analyses of sleep indicators in ADHD children have presented no significant differences between ADHD and typically developing (TD) children in total sleep time, wake after sleep onset, and stage shift (De Crescenzo et al., 2016; Díaz-Román, Hita-Yáñez, & Buela-Casal, 2016; Liang, Qiu, & Li, 2023). However, these meta-analyses have several limitations, such as uneven gender distribution in samples (male-dominated) and an insufficient number of included studies, which may affect the generalizability of the research conclusions. Thus, it is imperative to expand the sample size and further investigate the differences in these sleep metrics between ADHD and TD children.

In addition to the aforementioned indicators, sleep efficiency and sleep latency are also key sleep indicators. Sleep efficiency is defined as the ratio of actual sleep time to total time spent in bed, while sleep latency refers to the time it takes to fall asleep after getting into bed to prepare for sleep. Current research findings on sleep efficiency and sleep latency in ADHD children are inconsistent. Cortese, Faraone, Konofal, and Lecendreux (2009) found no significant difference in sleep efficiency between ADHD and TD children, but Hvolby, Jørgensen, and Bilenberg (2008) reported that the sleep latency of ADHD children was significantly longer than that of the TD children. In contrast, Díaz-Román et al. (2016) reported significant differences in sleep efficiency between ADHD and TD children, but found no significant differences in sleep latency. These inconsistent research findings may arise from multiple contributing factors: first, variations in evaluation criteria for research outcomes; second, disparities in the number of studies included; additionally, differences in sample composition. Given the significant impact of sleep efficiency and sleep latency on the sleep quality of ADHD children, and the inconsistent conclusions from previous studies, further investigation of these indicators is warranted.

The Awakening Index, which shows how often someone wakes up during the night, is very important for assessing and improving sleep quality. However, previous meta-analyses have not focused on an in-depth analysis of the Awakening Index. Therefore, this study will prioritize the Awakening Index as one of the key analysis indicators to fill this research gap.

When examining the differences in sleep characteristics between ADHD and TD children, it has been found that gender and age are not only important factors affecting sleep quality but may also play mediating and moderating roles in the differences in sleep indicators. For example, a meta-analysis by Lunsford-Avery, Krystal, and Kollins (2016) indicated that age is closely related to changes in sleep patterns, especially in children. Additionally, a study by Lindholm et al. (2024) found that boys with ADHD are more prone to sleep anxiety and nighttime awakenings than girls. However, most of these studies did not systematically compare the differences in sleep indicators between ADHD and TD children. Therefore, this study will conduct a comparative analysis of sleep indicators between the two groups, fully considering the impact of gender and age.

In summary, to address the aforementioned scientific questions, this study will employ a meta-analysis method, incorporating a large number of studies to systematically integrate and analyze existing sleep research data. The aim is to reveal the performance of ADHD children on key sleep indicators and to explore the impact of gender and age on these indicators. Through this study, we hope to provide evidence-based insights for the diagnosis, intervention, and treatment of ADHD, and to offer guidance for future research directions and clinical practice.

## Method

The protocol for this systematic review was preregistered on the Open Science Framework (OSF; Registration DOI: https://doi.org/10.17605/OSF.IO/QBP3H) and strictly adhered to the Preferred Reporting Items for Systematic Reviews and Meta-Analyses (PRISMA; Page et al., 2021) standards.

### Search strategy

We conducted a systematic review and meta-analysis of differences in sleep metrics between ADHD children and TD peers (The term “children” in this study refers to individuals aged 0–18 years.). Our search followed the Preferred Reporting Items for Systematic Reviews and Meta-Analyses guidelines in locating, reviewing, and excluding sources obtained from the search (Moher, Liberati, Tetzlaff, Altman, & Group, 2009). A coordinated search was conducted on major scholarly repositories, both national and international, including PubMed, Web of Science, and Google Scholar. A search algorithm based on a combination of the terms: (ADHD OR “attention deficit hyperactivity disorder”) AND (children OR adolescents) AND ("sleep time" OR “sleep cycle”) was used. Supplementary sources included documents identified from meta-analyses, research syntheses, review articles, and their reference lists.

### Selection criteria

Selection criteria were applied in the process of study retrieval to decide whether a study should be included in the meta-analysis. As a starting point for this process, two authors independently screened the titles and abstracts of each study to ensure that it met the basic inclusion criteria. When the fit was unclear, the corresponding author was consulted to ensure a valid inclusion decision.

The crucial inclusion criterion was that studies had to include sleep-related metrics that aligned with our operational definitions (e.g., total sleep time, sleep efficiency, sleep latency, wake after sleep onset, awakening index, stage shift). Equally important, studies had to report on participants with ADHD as well as age-matched TD controls. Notably, both ADHD and TD children had to be included in the sample, regardless of the group sizes (e.g., samples with many TD but few ADHD participants were also included). In light of the high rate of co-occurring conditions in ADHD, ADHD with any co-occurring sleep disorders were included to ensure the results of this review were generalizable to the ADHD population.

Studies were required to meet the other three inclusion criteria. First, the focus needed to be on participants under 18 years old. Second, the study had to be a quantitative study (including data from polysomnography, actigraphy, validated questionnaires, or quantifiable interview-based measures), with means and standard deviations reported to extract or calculate an effect size. Third, studies were included only if they were published in English in peer-reviewed journals.

**Figure 1.**
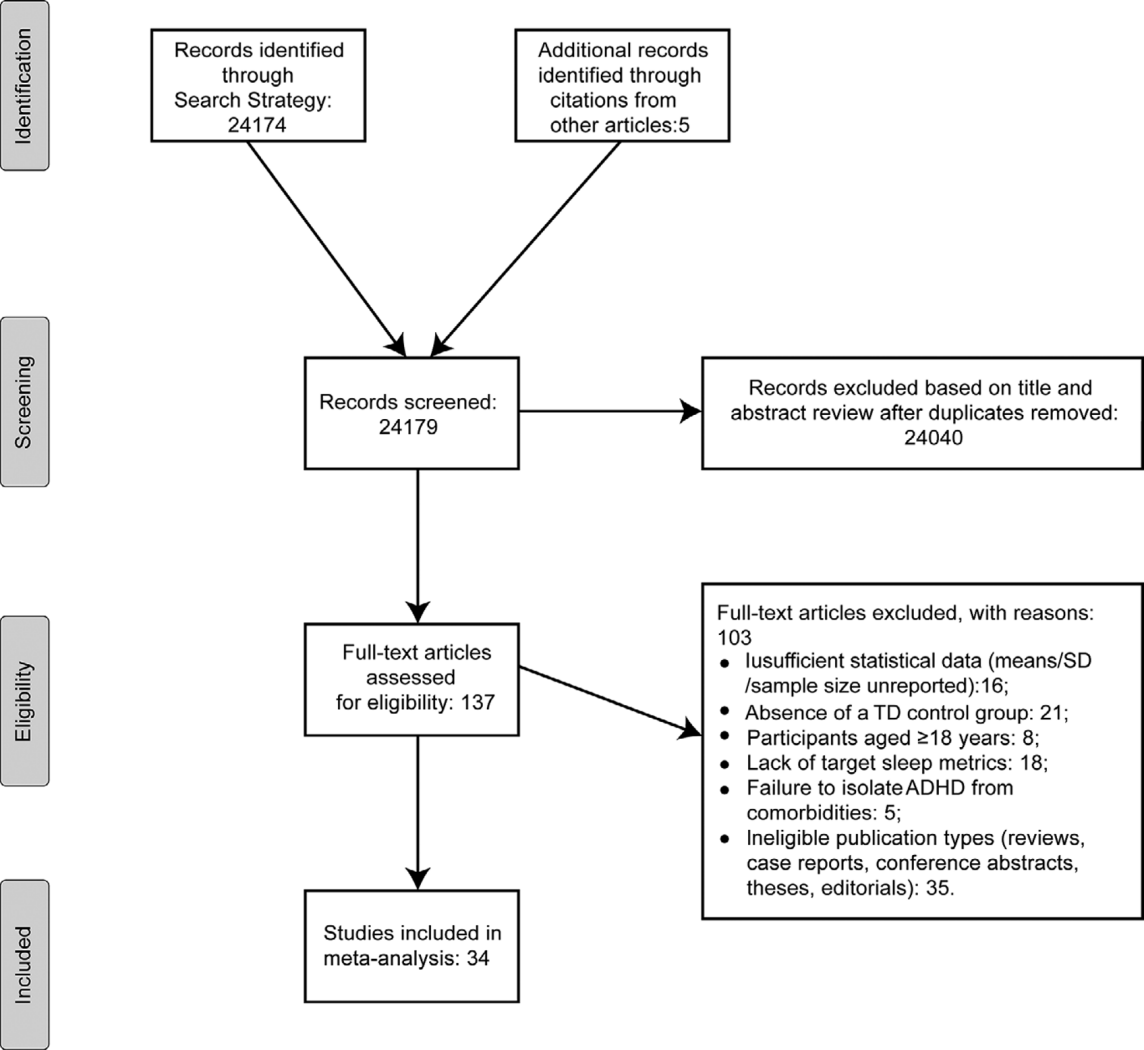
Flow chart summarizing the data search and inclusion process.

The search strategy resulted in 24,174 records identified (see Figure 1). Following removal of duplicates, titles and abstracts of these articles were assessed against eligibility criteria by two authors independently. Where inclusion or exclusion of a given article could not be determined by title or abstract alone, the two authors also assessed the full text. Full text screening occurred for 137 articles. Discrepancies in inclusion decisions were resolved through discussion, with the corresponding author consulted to ensure consensus was reached. Among the 137 studies available in the full-text review stage, 16 were excluded as they did not report sufficient statistics to calculate an effect size (comprising 3 studies excluded prior to pre-registration and 13 excluded during data extraction after pre-registration). The remaining full text exclusions were due to the absence of a TD control group (*n* = 21), participants aged 18 years or older (*n* = 8), lack of target sleep metrics (*n* = 18), failure to isolate ADHD from comorbidities (*n* = 5), or ineligibility of the publication type (e.g., reviews, case reports, conference abstracts, theses, editorials; *n* = 35). We itemized the specific reasons for the exclusion of all 103 articles screened out of the analysis in Supplementary Appendix 1. In total, 34 articles met the full inclusion criteria and underwent data extraction for this meta-analysis.

### Coding of variables

We ensured the validity of coding by first preparing a coding sheet where all relevant variables were listed. Two authors independently processed data extraction from 34 articles. The coded variables consisted of sample identification, publication year, sample size, mean age, and gender distribution (male/female) for both the ADHD and TD children. Additionally, clinical details were recorded, including ADHD subtype classifications (inattentive, hyperactive-impulsive, combined) and medication status (naïve, withdrawn, or currently medicated). Sleep-related measures were analyzed as continuous variables, with means and standard deviations calculated for each metric. If an article contained multiple experiments, they were analyzed as separate independent studies. To ensure reliability, two authors collaboratively reviewed the entire dataset, cross-checking coding accuracy and resolving inconsistencies through discussion with the corresponding author until consensus was reached. As a result of this multistep coding process, we are quite confident that the final data provided a valid reflection of the sampled literature.

### Data analysis

To investigate whether differences existed in sleep continuity between ADHD and TD, we examined indicators characterizing sleep continuity, including total sleep time and stage shift. Furthermore, we explored arousal phenomena disrupting sleep continuity using wake after sleep onset and awakening index as metrics of sleep fragmentation. Additionally, as sleep process effectiveness represents an essential indicator of sleep quality, we analyzed sleep efficiency and sleep latency as primary effect metrics to determine potential differences in sleep process effectiveness between ADHD and TD. The standardized mean difference (SMD) between the performance of each group was the measure of effect size. Furthermore, the small sample correction recommended by Hedges and Becker (1986) was applied to the effect sizes (nominally, Hedges’ *g*). For comprehensive synthesis, all meta-analytic results were computed by means of the metafor package in the R statistical software (version 4.3.3) (Viechtbauer, 2010), with model refinement guided by heterogeneity assessments as detailed below.

To validate model selection, a standard test of heterogeneity (Cochran’s test; DerSimonian & Laird, 1986) was used. *I* ^2^ of 25%, 50%, and 75% indicated small, medium, and large amounts of heterogeneity, respectively (Higgins & Thompson, 2002; Higgins, Thompson, Deeks, & Altman, 2003). According to these heterogeneity evaluations, the primary effects were ultimately analyzed using random-effects models when substantial heterogeneity was observed (*I* ^2^ ≥ 50%), whereas fixed-effects models were retained under low heterogeneity conditions (*I^2^* < 50%).

Since this study pooled data from multiple assessment types, it was necessary to clarify the impact of assessment type approaches on both the primary effects and heterogeneity. To examine whether results differed between each assessment type compared to the overall primary effects, we conducted subgroup meta-analyses stratified by assessment type. Furthermore, to determine whether the assessment type constituted a major source of heterogeneity, we performed meta-regression analyses.

Based on the results of primary effect analyses, regression analyses were conducted to investigate the relationships between demographic variables (gender, age, ADHD subtype distribution, and medication status) and differences in sleep metrics between the two groups. We further examined how demographic variables influenced the differences in primary sleep metrics using Hayes’ (Hayes, 2012) multiple moderation Model 3 and mediation Model 4, implemented through IBM SPSS Statistics 26 software with PROCESS v4.1.

To evaluate the risk of bias, as our study was a non-randomized study, the validity of the included studies was assessed using seven domain from the ROBINS-I Risk of Bias assessment tool (Sterne et al., 2016), including Confounding, Selection of participants, Classification of interventions, Deviations from intended interventions, Missing data, Measurement of outcomes, and Selection of the reported result. We formulated a set of risk of bias assessment criteria (see Supplementary Appendix 2). For this assessment, only the information that was actually reported in the papers was used, to ensure a consistent procedure across studies and to reduce the risk of bias based on what was reported and what was not. The assessment was carried out independently by two authors, with any disagreements resolved through discussion with the corresponding author.

To confirm the stability of the aforementioned analytical outcomes, consistent with Rücker, Carpenter, and Schwarzer (2011), numerous methods were employed to assess the data for publication bias and related small-study effects. A contour-enhanced funnel plot of each study’s effect size against its standard error was visually inspected. The trim-and-fill method (Duval & Tweedie, 2000) was applied to estimate the number of potentially missing studies and to impute effect sizes for any identified missing studies for each sleep metric. Egger’s test was also performed, where a non-significant *p*-value indicated the absence of publication bias.

Finally, to evaluate the quality of evidence, the Grading of Recommendations Assessment, Development and Evaluation (GRADE) system for grading evidence (Balshem et al., 2011) was employed. A set of evidence quality assessment criteria was formulated (see Supplementary Appendix 3). Similarly, the assessment was conducted independently by two authors and reached agreement by consulting the corresponding author.

## Results

This study systematically analyzed 34 articles (comprising 44 independent studies) to clarify the characteristic multidimensional sleep changes in ADHD children. A total of 2,239 ADHD children (77.0% male, mean age = 9.462 years) and 57,181 TD children (65.6% male, mean age = 9.472 years) were included in the analysis (see Supplementary Appendix 4). Sleep metrics were assessed through polysomnography, actigraphy, electroencephalography, parent-report, and self-report (see Supplementary Appendix 5). Through integration of meta-analysis, regression analysis, and mediation-moderation effect analyses, the study comprehensively mapped the landscape of ADHD-related sleep disturbances, which revealed persistent sleep dysregulation across multiple domains in ADHD children. These findings underscore the pervasiveness of sleep impairments in ADHD and provide new insights into potential mechanisms and moderating factors.

### Results of primary effect analyses

**Figure 2.**
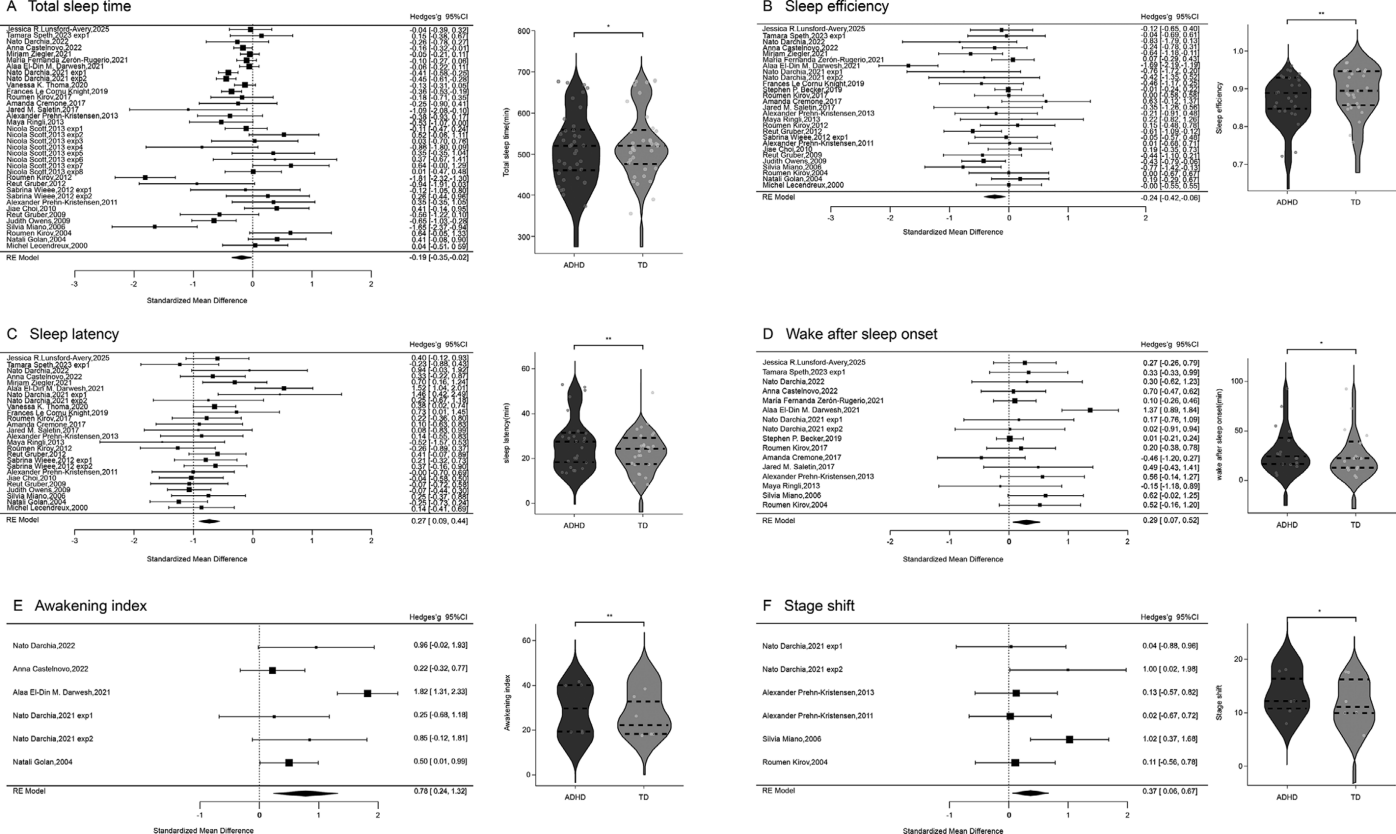
Forest plot of standardized mean difference (SMD) for meta-analysis dependent variable and violin plot for two-group comparison. *Note:* *p < 0.05; **p < 0.01.

*Regarding sleep continuity.* For total sleep time, substantial heterogeneity was observed across studies (*I* ^2^ = 87.5%, with all heterogeneity data presented in Supplementary Appendix 6), necessitating random-effects model analyses. The results showed that, compared with TD children, ADHD children exhibited significantly shorter total sleep time (ADHD: 518.918 minutes vs. TD: 525.873 minutes; SMD = −0.186, *p* = 0.027, with all meta-analytic results illustrated in Table 1 and Figure 2). In contrast, studies measuring stage shift demonstrated low heterogeneity (*I^2^* = 36.4%), enabling fixed-effects model analyses. The results identified a significant elevation in stage shift among ADHD children (ADHD: 12.967 vs. TD: 12.067; SMD = 0.367, *p* = 0.018).

**Table 1.** Primary meta-analyses results for sleep metrics (overall and stratified by assessment type)

Sleep metrics	Number of studies	Mean		SMD	95% CI
		ADHD	TD		
Total sleep time	36	518.918	525.837	–0.186*	–0.350, –0.021
Polysomnography	19	485.171	487.872	–0.151	–0.478, 0.175
Actigraphy	7	468.667	486.975	–0.317 **	–0.512, –0.122
Parent-report	8	644.875	654.750	–0.214 ***	–0.328, –0.101
Self-report	1	542.850	554.050	-	-
Electroencephalography	1	480.300	461.000	-	-
Sleep efficiency	27	0.881	0.892	–0.238 **	–0.417, –0.059
Polysomnography	19	0.900	0.909	–0.221	–0.458, 0.016
Actigraphy	7	0.830	0.849	–0.171 *	–0.325, –0.017
Electroencephalography	1	0.890	0.876	-	-
Sleep latency	26	27.685	23.448	0.269 **	0.094, 0.443
Polysomnography	18	26.669	23.656	0.220 *	0.004, 0.436
Actigraphy	6	31.668	22.982	0.470 *	0.086, 0.853
Self-report	1	31.150	21.050	-	-
Electroencephalography	1	18.600	24.900	-	-
Wake after sleep onset	16	31.883	28.657	0.294 *	0.069, 0.519
Polysomnography	11	23.155	18.494	0.412 **	0.119, 0.705
Actigraphy	4	53.881	52.545	-	-
electroencephalography	1	39.900	44.900	-	-
Awakening index	6	8.263	6.060	0.781 **	0.240, 1.322
Polysomnography	4	11.095	8.065	-	-
Actigraphy	2	2.600	2.050	-	-
Stage shift	6	12.967	12.067	0.367 *	0.064, 0.671
Polysomnography	4	13.425	12.650	-	-
Actigraphy	2	12.050	10.900	-	-

*Note.*

1. * *p* < 0.05, **p < 0.01, *** *p* < 0.001.

2. - means insufficient number of studies for meta-analysis

*Regarding sleep interruption.* The heterogeneity analyses of wake after sleep onset and awakening index demonstrated moderate heterogeneity across studies (*I^2^* = 56.4% and 73.7%, respectively), therefore, random-effects models were implemented. The results revealed significant elevations in both wake after sleep onset (ADHD 31.883 minutes vs. TD 28.657 minutes; SMD = 0.294, *p* = 0.011) and awakening index (ADHD 8.263 vs. TD 6.060; SMD = 0.781, *p* = 0.005).

*Regarding sleep process effectiveness.* Due to the moderate heterogeneity observed across studies examining sleep efficiency and sleep latency (*I* ^2^ = 62.6% and 54.6% respectively), random-effects models were implemented. The meta-analytic results showed that ADHD children exhibited notably reduced sleep efficiency (ADHD: 88.1% vs. TD: 89.2%; SMD = −0.238, *p* = 0.009), and significantly prolonged sleep latency (ADHD: 27.685 minutes vs. TD: 23.448 minutes; SMD = 0.269, *p* = 0.003).

The subgroup meta-analyses by assessment type demonstrated that all results were directionally consistent with the overall primary effects. All other outcomes exhibited statistically significant differences between ADHD and TD children (all *p* < 0.05), while sleep efficiency (polysomnography) demonstrated marginal significance (*p* = 0.068), and total sleep time (polysomnography) showed no significant (*p* = 0.363) group differences. Furthermore, meta-regression with assessment type as a covariate (see Supplementary Appendix 7) indicated that the assessment type approach was not a major source of heterogeneity (all *p* > 0.05).

Furthermore, primary effect meta-analyses were also conducted on time in bed, as well as the duration, proportion, and latency of N1 through REM sleep stages. However, no statistically significant result was found (*p* > 0.05 for all comparisons).

### Results of regression analyses

**Figure 3.**
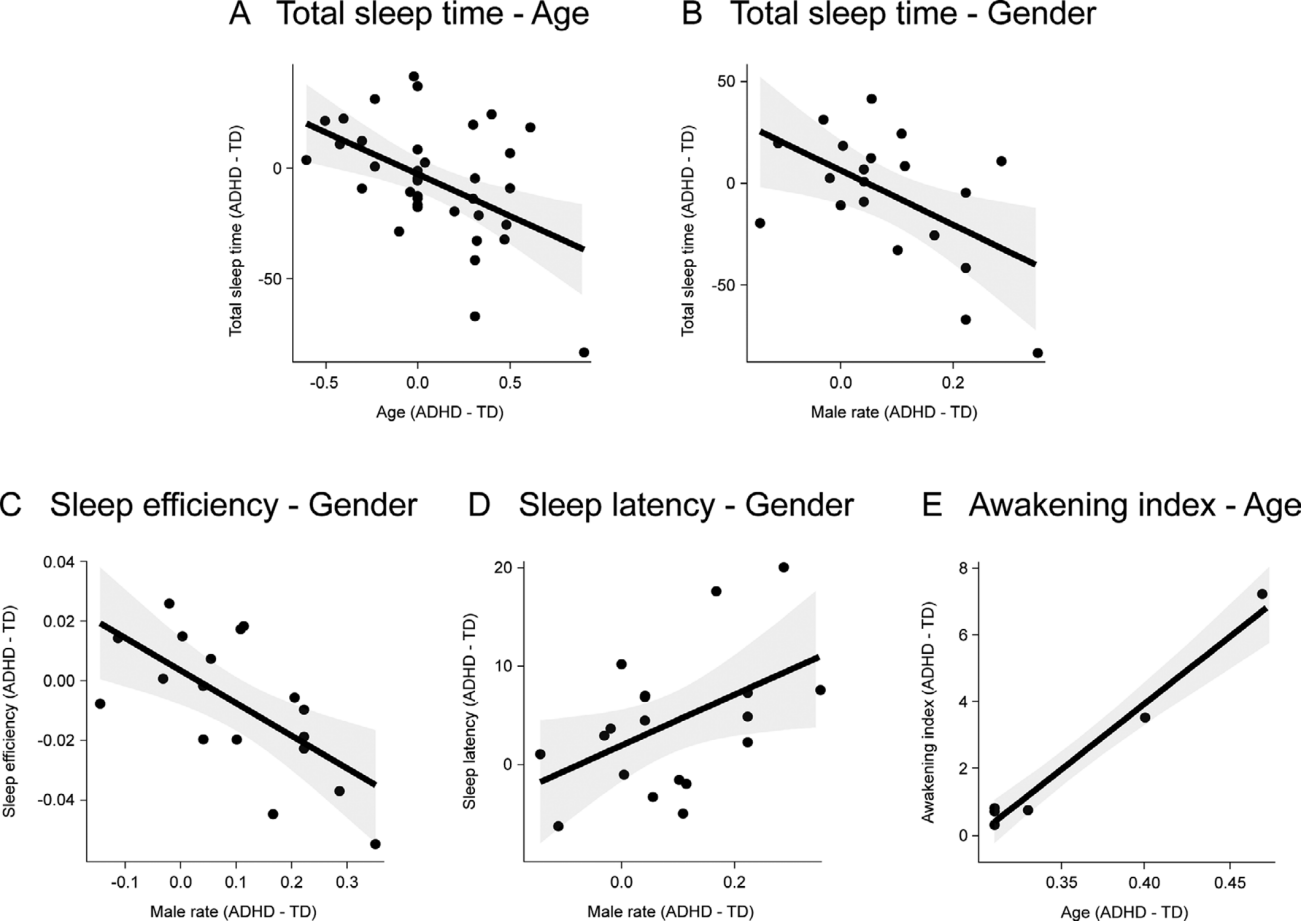
Scatter plot for regression analysis of demographic variables and sleep indicators. *Note:* Shade represents the 95% confidence bands.

The regression analyses revealed that gender composition and age had a significant impact on sleep metric abnormalities (see Figure 3). When the male rate was higher in the ADHD children, the difference in total sleep time between two groups widened (*β* = −0.549, *p* = 0.012), the decline in sleep efficiency intensified (*β* = −0.659, *p* = 0.003), and the prolongation of sleep latency became more pronounced (*β* = 0.476, *p* = 0.040). As age increased, the differences between the ADHD and TD children progressively widened, with the ADHD children exhibiting shorter total sleep time (*β* = 0.482, *p* = 0.001) and a significantly elevated awakening index (*β* = 0.993, *p* = 0.001).

Additionally, no significant effect of gender or age on wake after sleep onset was observed. Furthermore, no significant association was found between ADHD subtype distributions (inattentive, hyperactive-impulsive, combined) or medication status (medication-naïve, medication-discontinued, currently medicated) and sleep metrics (all *p* > 0.05).

### Results of moderation model analyses

**Figure 4.**
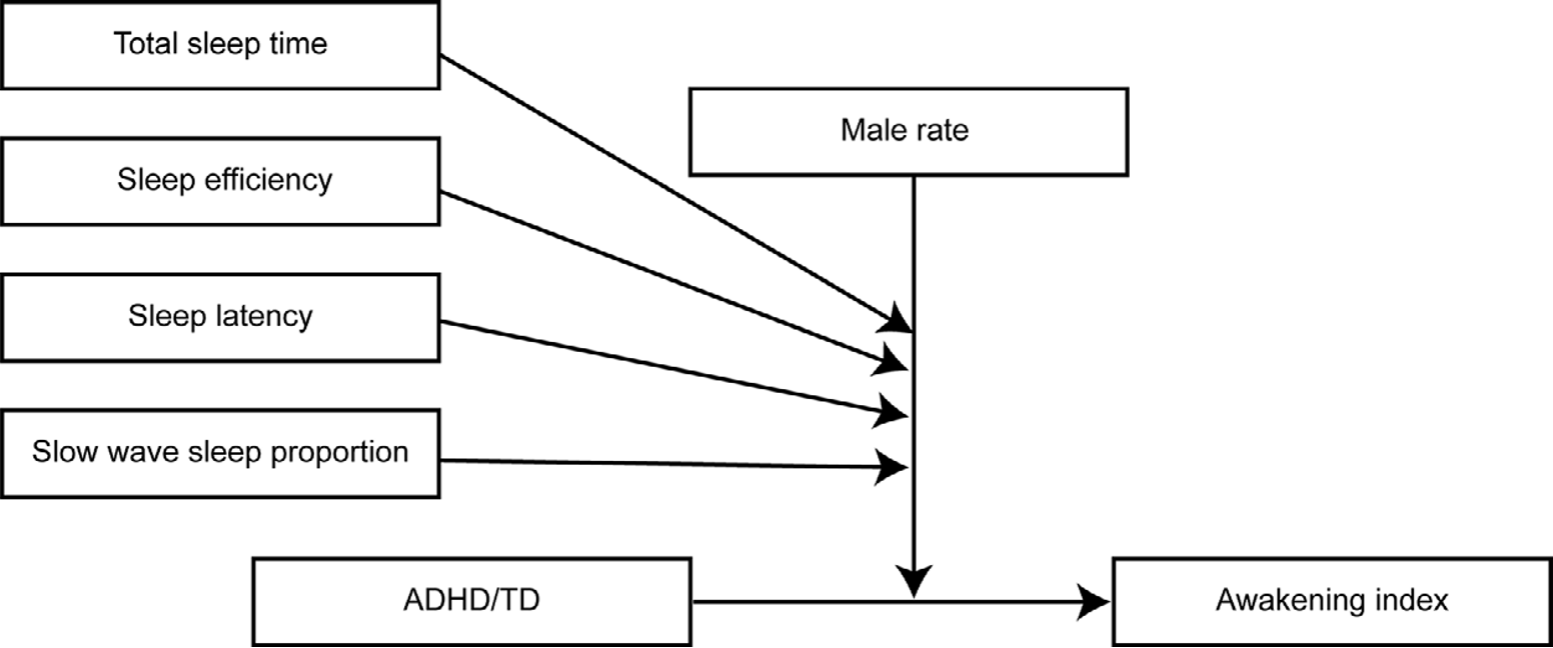
Multiple moderation model diagram for awakening index.

When awakening index served as the dependent variable, significant interactions were identified for ADHD/TD × male rate × sleep latency, ADHD/TD × male rate × sleep efficiency, ADHD/TD × male rate × total sleep time, and ADHD/TD × male rate × slow-wave sleep proportion (see Figure 4). The ADHD/TD × male rate × total sleep time interaction (*t* = -20.472, *p* = 0.002) revealed that in studies with medium-high male proportions, increased total sleep time was paradoxically associated with worsening awakening index in the ADHD children, whereas the TD children exhibited expected improvements. The ADHD/TD × male rate × sleep efficiency interaction (*t* = 10.128, *p* = 0.010) demonstrated that in studies with low to medium male proportions, enhanced sleep efficiency significantly reduced the awakening index in both groups. However, in studies with higher male proportions, improved sleep efficiency unexpectedly exacerbated the awakening index in the ADHD children, while the TD children maintained the anticipated reduction. The ADHD/TD × male rate × sleep latency interaction (*t* = 23.141, *p* = 0.002) indicated that across varying male proportions, prolonged sleep latency consistently attenuated awakening index to differing degrees. The ADHD/TD × male rate × slow-wave sleep proportion interaction (*t* = −7.430, *p* = 0.018) displayed that at high male proportions, increased slow-wave sleep significantly ameliorated awakening index in the ADHD children. Conversely, under low-medium male proportions, the ADHD children showed that elevated slow-wave sleep proportions unexpectedly aggravated awakening index deterioration.

Subsequently, when stage shift served as the dependent variable, these analyses demonstrated that the ADHD/TD × time in bed × sleep efficiency interaction reached statistical significance (*t* = 2.878, *p* = 0.045, see Supplementary Appendix 8). The results revealed that under short time-in-bed conditions, improved sleep efficiency showed no significant ameliorative effects on stage shift. Conversely, the combination of extended time in bed with elevated sleep efficiency appeared to promote buffering against excessive stage shift.

No other significant moderation model was identified (all *p* > 0.05).

### Results of mediation model analyses

The mediation analyses demonstrated that slow wave sleep latency exerted a full mediation effect between ADHD/TD classification and increased awakening index (see Supplementary Appendix 9). ADHD/TD indirectly contributed to elevated awakening index through prolonged slow wave sleep latency (*t* = 3.738, *p* = 0.065, marginal significance), which subsequently mediated increased awakening index (*t* = -7.133, *p* = 0.089, marginally significant). The direct path from ADHD/TD to the awakening index was not significant (*t* = 1.172, *p* = 0.450).

No other significant or marginally significant mediation model was identified (all *p* > 0.1).

### Risk of bias assessment

The risk of bias assessment of the included studies based on the ROBINS-I was shown in Supplementary Appendix 2. Nineteen studies were rated at low risk (19/44, 43.2%), and 25 studies met criteria for moderate risk of bias (25/44, 56.8%). Consequently, the overall risk of bias was not substantial.

### Publication bias analyses

Initially, separate funnel plots were generated for total sleep time, sleep efficiency, sleep latency, wake after sleep onset, awakening index, and stage shift (see Supplementary Appendix 10). These plots showed a fairly symmetrically distributed pattern of effect sizes for all sleep metrics. Second, no missing studies were identified for total sleep time, sleep efficiency, sleep latency, or stage shift using the trim-and-fill method. However, five missing studies were detected for wake after sleep onset and one missing study was identified for the awakening index (see Supplementary Appendix 11). The adjusted standardized mean difference (SMD) and 95%CI for these metrics are summarized in Appendix 12. Third, Egger’s test was conducted on the overall analyses for each sleep metric and was nonsignificant for all sleep metrics. Synthesizing across metrics of publication bias, wake after sleep onset showed potential risk of publication bias, while results for other metrics remained robust, with all publication bias test results are presented in Supplementary Appendix 12.

### Quality of evidence assessment

According to GRADE guidelines, our baseline grading for the evidence commenced from the low-quality level. Consequently, all included studies were awarded low or very low ratings (see Supplementary Appendix 13), though care should be taken when interpreting results given the inconsistency between studies. Such inconsistency was unsurprising given the broad heterogeneity of the ADHD spectrum.

## Discussion

This study aimed to systematically explore the sleep characteristics of ADHD children and TD children, with a particular focus on the moderating roles of gender and age. The results of this study indicated that ADHD children have significant sleep deficits in several key metrics. They have shorter total sleep time, lower sleep efficiency, longer sleep latency, and a higher awakening index compared to TD children. These findings highlight the pervasive nature of sleep disturbances in ADHD and underscore the importance of considering sleep issues in the broader context of ADHD management.

To more accurately verify the clinical significance, this study has re-examined the limitations of previous meta-analyses through methodological optimization. Previous meta-analyses (Díaz-Román et al., 2016; Liang et al., 2023) failed to find significant differences between ADHD children and TD children in total sleep time, wake after sleep onset, and sleep stage transitions. However, their conclusions were limited by the use of single measurement methods and an insufficient number of included studies. By increasing the variety of measurement methods and expanding the sample size, this study has re-validated the differences in these sleep indicators. The results showed that the total sleep time in the ADHD children was significantly shortened, wake after sleep onset was significantly prolonged, and the frequency of sleep stage transitions was significantly increased. The contradictions between these findings and previous conclusions may originate from the following improvements in this study: First, when calculating the primary effect, this study used only the mean and standard deviation as key statistical metrics. Compared to the effect size conversion steps commonly used in other studies, this method effectively reduced potential statistical errors and improved the accuracy of the results. Second, while previous studies mostly relied on subjective reports (such as questionnaires) or single objective indicators (such as actigraphy), this study integrated multimodal data (polysomnography, electroencephalogram), which enhanced the measurement precision of dynamic indicators such as sleep stage transitions. Third, this study controlled for the interference of group differences through heterogeneity tests and moderator effect analyses (such as gender and age) and used a random-effects model (*I* ^2^ ≥ 50%) to reduce the impact of methodological heterogeneity. Previous studies may have underestimated the effect size by ignoring such moderator variables or using a fixed-effects model. Fourth, to ensure the reliability and robustness of the study results, we have systematically conducted extensive publication bias tests in this study. By employing various statistical methods (such as funnel plot analysis, Trim and Fill method, and Egger’s test), we comprehensively assessed the impact of potential publication bias on the study results, enhancing the scientific nature and credibility of the conclusions.

Notably, considering the previous conflicting conclusions regarding sleep efficiency and latency (Cortese et al., 2009; Díaz-Román et al., 2016), this study found that the sleep latency of ADHD children is significantly prolonged, and their sleep efficiency is significantly reduced, which supports some of the conclusions drawn by Cortese et al. (2009). However, this study further revealed that gender composition is a key moderator variable. For example, when the proportion of males is high, an increase in sleep efficiency may exacerbate awakening disorders, suggesting that gender-specific mechanisms may explain the differences in previous studies. This phenomenon may be related to the higher sensitivity of the dopaminergic system in males (Makino et al., 2024), and the imbalance of neurotransmitters may weaken the regulation of sleep homeostasis. At the same time, when the proportion of males is high, the longer the sleep latency, the lower the arousal index. This may be because a prolonged sleep latency may mean that ADHD children need more time to enter deep sleep after falling asleep. Due to the extended time to fall asleep, they may spend more time in deep sleep within a limited sleep duration to compensate for sleep quality, thereby reducing the number of awakenings.

In addition to sleep efficiency and latency, this study has also filled the research gap in another key sleep indicator—arousal index, providing a new direction for clinical interventions. This study has found that the arousal index in the ADHD children is significantly elevated and that this indicator is fully mediated by slow-wave sleep latency. This result not only fills the gap in previous meta-analyses but also provides a new target for clinical assessment. For example, intervening in slow-wave sleep latency may improve sleep continuity by reducing the frequency of awakenings. Moreover, the gender-specific moderation of the arousal index (such as limited intervention effects when the proportion of males is high) suggests the need for personalized intervention strategies, with particular attention to the abnormal neurodevelopmental rhythms in male ADHD children.

In this study, we have not only discovered differences in various sleep indicators between ADHD and TD children but also identified the moderating and mediating effects of gender and age. The above conclusions have elaborated on some of these moderating and mediating effects, and the multiple-moderator model presented in Figure 4 provides an important perspective for understanding the complex mechanisms underlying sleep problems in ADHD children. This model reveals how the gender ratio moderates the impact of sleep latency, sleep efficiency, total sleep time, and slow-wave sleep proportion on the arousal index. Specifically, the moderating effect of total sleep time indicates that the impact of prolonged total sleep time on the arousal index varies significantly depending on the gender ratio. For example, in the children with ADHD a higher proportion of males, an extended total sleep time actually worsens the arousal index. This finding may be related to the abnormal neurodevelopmental rhythms in male ADHD children, suggesting that simply extending sleep duration may not be sufficient to overcome sleep continuity obstacles (El-Monshed et al., 2024). Second, the moderating effect of slow-wave sleep proportion shows that the impact of increased slow-wave sleep proportion on the arousal index also varies significantly depending on the gender ratio. For example, among ADHD children, those with a higher proportion of males show a significant improvement in the arousal index when the proportion of slow-wave sleep is increased. This result suggests that the neuroprotective effect of slow-wave sleep is more pronounced in male ADHD children, possibly due to gender differences in the dopaminergic system (Zhao, Li, Chen, & Lei, 2020). In addition, the regulatory model of time in bed reveals the dynamic balance between time in bed and sleep efficiency through a non-linear moderating effect. The combined effect of prolonged time in bed and improved sleep efficiency promotes the maintenance of sleep-wake homeostasis and reduces sudden sleep stage transitions (Yang et al., 2025). This regulatory effect provides important metrics for clinical interventions, emphasizing the need to achieve a dynamic balance between sleep duration and sleep structure integrity during sleep optimization. Moreover, this study has used regression analysis to reveal a significant association between age and total sleep time and arousal index. However, further analysis showed that age does not have a significant moderating effect on total sleep time and arousal index. This result indicates that although age has a certain impact on sleep quality, this impact does not occur through the mechanism of regulating other variables.

### Limitations

Despite the progress made in the analysis of multidimensional sleep indicators, the following limitations must be acknowledged: First, the subgroup analysis was underpowered. ADHD subtypes (inattentive, hyperactive-impulsive, combined) and medication status (unmedicated, medication withdrawal, medicated) did not show significant effects, which may be attributed to the insufficient sample size. Future studies need to expand subgroup samples to verify potential differences. Second, confounding variables (such as sociocultural factors) that may influence sleep behavior could not be ruled out. Third, the effect of slow-wave latency in the mediation model was only marginally significant, which may reflect measurement errors or the complexity of the neural mechanisms. Combining fMRI technology to explore the activity of slow-wave-related brain regions (such as the prefrontal-thalamic circuit) can deepen the understanding of the underlying mechanisms.

### Future research directions

Based on the above findings and limitations, future research can be advanced in three directions: First, develop gender- and developmental stage-specific intervention plans. For example, for male children with ADHD, the efficacy of theta/beta ratio (TBR) and sensorimotor rhythm (SMR) neurofeedback can be tested (Arns, Feddema, & Kenemans, 2014), while female children with ADHD may benefit from cognitive-behavioral therapy to shorten sleep latency. Second, integrate multimodal neuroimaging with sleep monitoring. Capturing the dynamic brain network during slow-wave sleep using fMRI (such as the default mode network) can reveal the neural circuit mechanisms underlying sleep disorders in ADHD (Stevner et al., 2019). Third, future research should explore new non-pharmacological intervention pathways. Transcranial magnetic stimulation (TMS) to modulate neural oscillations (Thut et al., 2011) or digital sleep management platforms (such as AI-based sleep stage feedback) may break through the bottlenecks of traditional behavioral interventions.

## Conclusion

This study systematically analyzed the sleep-related characteristics of ADHD children. The findings indicated that, in comparison with TD children, ADHD children have significantly shorter total sleep time, longer sleep latency, and a higher awakening index. Gender and age play significant moderating roles in these differences. Male ADHD children are more sensitive to sleep-metric changes, possibly due to gender-related dopaminergic-system differences. Slow-wave sleep is a potential mediator and moderator for the increased awakening index, indicating its possible intervention-target potential. These findings highlight the need for interventions addressing sleep problems in ADHD children and suggest future research directions.

## Supporting information

Xian et al. supplementary material Xian et al. supplementary material

## Supplementary material

The supplementary material for this article can be http://doi.org/10.1017/S0033291725102158.
