# Supplementary material for: Sleep dysregulation in ADHD children: a systematic review and meta-analysis
Source: Psychol Med. 2025 Oct 28;55:e321. doi: 10.1017/S0033291725102158 (PMC13054914; doi:10.1017/S0033291725102158)
Supplement: Xian et al. supplementary material [file S0033291725102158sup001.docx]

**Sleep Dysregulation in ADHD Children: A Systematic Review and Meta-Analysis**

**Overview Appendices**

| In-Text Citation | Content | Page number |
| --- | --- | --- |
| Appendix 1 | Reasons for article exclusion | 2 |
| Appendix 2 | Risk of Bias Assessment Framework | 5 |
| Appendix 3 | GRADE Evidence Quality Assessment | 6 |
| Appendix 4 | Demographic Background Information of Participants across Studies for Meta-Analysis | 7 |
| Appendix 5 | Primary Sleep Metrics, Assessment Type, and Key Outcomes across Studies for Meta-Analysis | 9 |
| Appendix 6 | Overview of Heterogeneity Data for Sleep Metrics in Meta-Analysis | 12 |
| Appendix 7 | Results of the meta-regression with assessment type as a covariate | 13 |
| Appendix 8 | Multiple Moderation Model Diagram for Stage Shift | 14 |
| Appendix 9 | Mediation Model Diagram for Awakening Index | 15 |
| Appendix 10 | Contour-Enhanced Funnel Plots of Included Studies’ Effect Sizes | 16 |
| Appendix 11 | Funnel Plot of Imputed and Included Studies’ Effect Sizes Investigating Wake after Sleep Onset and Awakening Index | 17 |
| Appendix 12 | Overview of Publication Bias Evaluation Results | 18 |
| Appendix 13 | Rating of Evidence Quality for Sleep Metrics | 19 |

**Appendix 1 Reasons for article exclusion**

| Study | Reasons for exclusion |
| --- | --- |
| [Marit Boxum, 2024](https://link.springer.com/article/10.1007/s10484-024-09649-y#auth-Marit-Boxum-Aff1-Aff2) | lneligible publication types (reviews, case reports, conference abstracts, theses, editorials) |
| Antonio F. Pagán, 2024 | Absence of a TD control group |
| Li Xiao, 2024 | Absence of a TD control group |
| Samuele Cortese, 2024 | lneligible publication types (reviews, case reports, conference abstracts, theses, editorials) |
| Claudio Arias-Mera, 2023 | lneligible publication types (reviews, case reports, conference abstracts, theses, editorials) |
| Craig A. Sidol, 2023 | Absence of a TD control group |
| Finja Marten, 2023 | Absence of a TD control group |
| I. Larsson, 2023 | lneligible publication types (reviews, case reports, conference abstracts, theses, editorials) |
| Stephany Fulda, 2023 | lneligible publication types (reviews, case reports, conference abstracts, theses, editorials) |
| Maria Silvia Saccani, 2022 | Lack of target sleep metrics |
| Manuel Munz, 2022 | Lack of target sleep metrics |
| Anna Ricci, 2022 | Failure to isolate ADHD from comorbidities |
| [Nicholas A Donnelly, 2022](https://elifesciences.org/articles/75482#xc7bc3dbf) | Failure to isolate ADHD from comorbidities |
| [Anna Ricci, 2022](https://academic.oup.com/sleep/article/45/3/zsab287/javascript:;) | Failure to isolate ADHD from comorbidities |
| [Xiao Liang, 2022](https://loop.frontiersin.org/people/1377395) | Lack of target sleep metrics |
| E. Martinez-Cayuelas, 2022 | Absence of a TD control group |
| Huimei Yin, 2022 | Absence of a TD control group |
| Upasana Bondopadhyay, 2022 | lneligible publication types (reviews, case reports, conference abstracts, theses, editorials) |
| Angela Ann Joseph, 2022 | lneligible publication types (reviews, case reports, conference abstracts, theses, editorials) |
| Marina Xavier Carpena, 2022 | lnsufficient statistical data (means/SD/sample size unreported) |
| Maya K. Malkani, 2022 | lneligible publication types (reviews, case reports, conference abstracts, theses, editorials) |
| Noelia Ruiz-Herrera, 2021 | Absence of a TD control group |
| Craig B. H. Surman, 2021 | Participants aged ≥18 years. |
| Carlo Biancardi, 2021 | lneligible publication types (reviews, case reports, conference abstracts, theses, editorials) |
| Kazutaka Ohi, 2021 | lnsufficient statistical data (means/SD/sample size unreported) |
| Samantha Scholes, 2020 | lneligible publication types (reviews, case reports, conference abstracts, theses, editorials) |
| Stéphanie Bioulac, 2020 | lneligible publication types (reviews, case reports, conference abstracts, theses, editorials) |
| Amanda Cremone-Caira, 2020 | Lack of target sleep metrics |
| Maria Korman, 2020 | Lack of target sleep metrics |
| Alexander Prehn-Kristensen, 2020 | Lack of target sleep metrics |
| Alexander Prehn-Kristensen, 2020 | Lack of target sleep metrics |
| Christina A. Martin, 2020 | Absence of a TD control group |
| Joshua M. Langberg, 2020 | Absence of a TD control group |
| Jane Nikles, 2020 | lneligible publication types (reviews, case reports, conference abstracts, theses, editorials) |
| Natalie L Phillips, 2020 | lneligible publication types (reviews, case reports, conference abstracts, theses, editorials) |
| Chun Shen, 2020 | lnsufficient statistical data (means/SD/sample size unreported) |
| Xianchen Liu, 2020 | lnsufficient statistical data (means/SD/sample size unreported) |
| Serena Scarpelli, 2019 | lneligible publication types (reviews, case reports, conference abstracts, theses, editorials) |
| [Silvia Miano, 2019](https://www.sciencedirect.com/author/6603458559/silvia-miano) | Lack of target sleep metrics |
| Nicole Papadopoulos, 2019 | Failure to isolate ADHD from comorbidities |
| Joshua M. Langberg, 2019 | lnsufficient statistical data (means/SD/sample size unreported) |
| Marina Xavier Carpena, 2019 | Participants aged ≥18 years. |
| A. N. Coogan, 2019 | Lack of target sleep metrics |
| Sang-Hwa Lee, 2019 | lneligible publication types (reviews, case reports, conference abstracts, theses, editorials) |
| Speth- Tamara, 2019 | lneligible publication types (reviews, case reports, conference abstracts, theses, editorials) |
| Jared M. Saletin, 2019 | lneligible publication types (reviews, case reports, conference abstracts, theses, editorials) |
| Stephen V. Faraone, 2019 | lneligible publication types (reviews, case reports, conference abstracts, theses, editorials) |
| [F. De Dea, 2018](https://ieeexplore.ieee.org/author/37086492019) | lneligible publication types (reviews, case reports, conference abstracts, theses, editorials) |
| Lian Tong, 2018 | Absence of a TD control group |
| Stephen P. Becker, 2018 | Absence of a TD control group |
| Ibtihal Siddiq Abdelgadir, 2018 | lneligible publication types (reviews, case reports, conference abstracts, theses, editorials) |
| Dafna Wajszilber, 2018 | lnsufficient statistical data (means/SD/sample size unreported) |
| Caroline Lustenberger, 2017 | Failure to isolate ADHD from comorbidities |
| Christian D. Wiesner, 2017 | Lack of target sleep metrics |
| Alexander Prehn-Kristensen, 2017 | Lack of target sleep metrics |
| James G. Waxmonsky, 2017 | Lack of target sleep metrics |
| E Sciberras, 2017 | Absence of a TD control group |
| Julia Grünwald, 2017 | Absence of a TD control group |
| Andrew N. Coogan, 2017 | lneligible publication types (reviews, case reports, conference abstracts, theses, editorials) |
| Cremone, Amanda, 2017 | lneligible publication types (reviews, case reports, conference abstracts, theses, editorials) |
| Shweta Anand, 2017 | lneligible publication types (reviews, case reports, conference abstracts, theses, editorials) |
| Stephen P. Becker, 2017 | lnsufficient statistical data (means/SD/sample size unreported) |
| Rackeb Tesfaye, 2017 | lnsufficient statistical data (means/SD/sample size unreported) |
| Niall M. McGowan, 2016 | Participants aged ≥18 years. |
| Felipe Kalil Neto, 2016 | Absence of a TD control group |
| Heather E. Schneider, 2016 | lnsufficient statistical data (means/SD/sample size unreported) |
| Margaret D. Weiss, 2015 | lneligible publication types (reviews, case reports, conference abstracts, theses, editorials) |
| Katherine M. Kidwell, 2015 | lneligible publication types (reviews, case reports, conference abstracts, theses, editorials) |
| [Allan Hvolby, 2015](https://link.springer.com/article/10.1007/s12402-014-0151-0#auth-Allan-Hvolby-Aff1) | lnsufficient statistical data (means/SD/sample size unreported) |
| Stephens, Robyn J., 2013 | Participants aged ≥18 years. |
| Karen L. Gamble, 2013 | Participants aged ≥18 years. |
| Sun Young Rosalia Yoon, 2013 | Participants aged ≥18 years. |
| [Samuele Cortese, 2013](https://www.sciencedirect.com/author/8651678100/samuele-cortese) | Absence of a TD control group |
| Jessica R. Barrett, 2013 | lneligible publication types (reviews, case reports, conference abstracts, theses, editorials) |
| Seyed-Ali Mostafavi, 2012 | Lack of target sleep metrics |
| Sun Young Rosalia Yoon, 2012 | lneligible publication types (reviews, case reports, conference abstracts, theses, editorials) |
| Jamie Cassoff, 2012 | lneligible publication types (reviews, case reports, conference abstracts, theses, editorials) |
| Martijn Arns, 2012 | lneligible publication types (reviews, case reports, conference abstracts, theses, editorials) |
| Alexander Prehn-Kristensen, 2011 | Lack of target sleep metrics |
| Karen Spruyt, 2011 | Absence of a TD control group |
| Sarah Ironside, 2010 | Lack of target sleep metrics |
| Oliviero Bruni, 2010 | lneligible publication types (reviews, case reports, conference abstracts, theses, editorials) |
| [Rosalia Silvestri, 2009](https://www.sciencedirect.com/author/7005023667/rosalia-c-silvestri) | Lack of target sleep metrics |
| Reut Gruber, 2009 | lneligible publication types (reviews, case reports, conference abstracts, theses, editorials) |
| Judith A Owens, 2009 | lneligible publication types (reviews, case reports, conference abstracts, theses, editorials) |
| Arthur S. Walters, 2008 | lneligible publication types (reviews, case reports, conference abstracts, theses, editorials) |
| Kristiaan B. Van der Heijden, 2007 | Absence of a TD control group |
| Roumen Kirov, 2007 | lnsufficient statistical data (means/SD/sample size unreported) |
| Avi Sadeh, 2006 | lneligible publication types (reviews, case reports, conference abstracts, theses, editorials) |
| Margaret D. Weiss, 2006 | Absence of a TD control group |
| Samuele Cortese, 2006 | lneligible publication types (reviews, case reports, conference abstracts, theses, editorials) |
| Alexandra Philipsen, 2005 | Participants aged ≥18 years. |
| Mairav Cohen-Zion, 2004 | lnsufficient statistical data (means/SD/sample size unreported) |
| Valerie Mclaughlin Crabtree, 2003 | Absence of a TD control group |
| Ruth Segal, 2000 | Participants aged ≥18 years. |
| Norbert Börger, 2000 | Lack of target sleep metrics |
| Judith A.Owens, 2000 | lnsufficient statistical data (means/SD/sample size unreported) |
| Mark A. Stein, 1999 | Absence of a TD control group |
| Aliza Ring, 1998 | lnsufficient statistical data (means/SD/sample size unreported) |
| Penny Corkum, 1998 | lnsufficient statistical data (means/SD/sample size unreported) |
| [Yaron Dagan, 1997](https://onlinelibrary.wiley.com/authored-by/DAGAN/YARON) | lnsufficient statistical data (means/SD/sample size unreported) |
| Jo-Ann B. Hoeppner, 1996 | Absence of a TD control group |
| [Steven P. Cuffe, 1994](https://link.springer.com/article/10.1007/BF02234689#auth-Steven_P_-Cuffe-Aff1) | Lack of target sleep metrics |

**Appendix 2 Risk of Bias Assessment Framework**

| Bias Domain | Key Assessment Items | | Jessica R. Lunsford-Avery,2025 | Blanka Vojnits, 2024 | Pınar Özbudak,2024 | Tamara Speth,2023 exp1 | Tamara Speth,2023 exp1 | Nato Darchia,2022 | Anna Castelnovo,2022 | Mirjam Ziegler,2021 | María Fernanda Zerón‑Rugerio,2021 | Alaa El-Din M. Darwesh,2021 | Nato Darchia,2021 exp1 | Nato Darchia,2021 exp2 | Vanessa K. Thoma,2020 | Federica De Dea,2019 | Yoshihiko Saito,2019 | Frances Le Cornu Knight,2019 | Stephen P. Becker,2019 | Roumen Kirov,2017 | Amanda Cremone,2017 | Jared M. Saletin,2017 | Gulcin Akinci,2015 | Alexander Prehn-Kristensen,2013 | Maya Ringli,2013 | Nicola Scott,2013 exp1 | Nicola Scott,2013 exp2 | Nicola Scott,2013 exp3 | Nicola Scott,2013 exp4 | Nicola Scott,2013 exp5 | Nicola Scott,2013 exp6 | Nicola Scott,2013 exp7 | Nicola Scott,2013 exp8 | Roumen Kirov,2012 | Reut Gruber,2012 | Sabrina Wieee,2012 exp1 | Sabrina Wieee,2012 exp2 | Alexander Prehn-Kristensen,2011 | Jiae Choi,2010 | Reut Gruber,2009 | Judith Owens,2009 | Silvia Miano,2006 | Roumen Kirov,2004 | Natali Golan,2004 | Michel Lecendreux,2000 | Penny Corkum,1999 |
| --- | --- | --- | --- | --- | --- | --- | --- | --- | --- | --- | --- | --- | --- | --- | --- | --- | --- | --- | --- | --- | --- | --- | --- | --- | --- | --- | --- | --- | --- | --- | --- | --- | --- | --- | --- | --- | --- | --- | --- | --- | --- | --- | --- | --- | --- | --- |
| **Confounding** | Key confounders controlled? | Age | Y | W | Y | Y | Y | Y | Y | Y | Y | Y | Y | Y | Y | Y | Y | Y | Y | Y | Y | Y | Y | Y | Y | Y | Y | Y | Y | Y | Y | Y | Y | Y | Y | W | W | Y | Y | Y | Y | W | Y | Y | Y | W |
|  |  | Gender | N | W | Y | W | W | N | N | N | Y | Y | N | N | Y | W | W | W | Y | Y | Y | Y | Y | Y | Y | W | W | W | W | W | W | W | W | Y | Y | W | W | N | Y | Y | N | W | W | Y | Y | W |
|  |  | IQ | W | W | W | N | N | Y | W | N | W | Y | Y | Y | Y | W | W | W | W | Y | W | N | Y | Y | Y | W | W | W | W | W | W | W | W | Y | Y | W | W | Y | Y | Y | W | W | Y | W | W | W |
|  |  | Sleep environment | Y | N | Y | Y | Y | Y | Y | N | W | Y | Y | Y | N | W | W | N | N | Y | Y | Y | Y | N | Y | W | W | W | W | W | W | W | W | Y | N | N | N | N | W | N | N | Y | Y | Y | Y | W |
|  |  | Comorbid sleep disorders | Y | W | Y | Y | Y | Y | Y | W | W | W | Y | Y | N | W | W | Y | Y | Y | Y | Y | N | Y | Y | N | N | N | N | N | N | N | N | Y | Y | Y | Y | Y | N | N | Y | Y | Y | Y | Y | W |
|  |  | Psychiatric comorbidities | Y | W | Y | Y | Y | Y | Y | Y | Y | Y | Y | Y | Y | Y | W | Y | Y | N | Y | Y | Y | Y | Y | N | N | N | N | N | N | N | N | Y | Y | Y | Y | Y | Y | Y | Y | Y | Y | N | Y | N |
| Judgment | | | moderate | serious | low | moderate | moderate | low | moderate | serious | moderate | low | low | low | moderate | serious | serious | moderate | moderate | low | low | low | low | low | low | serious | serious | serious | serious | serious | serious | serious | serious | low | low | serious | serious | moderate | moderate | moderate | moderate | moderate | low | low | low | critical |
| **Selection of participants** | Both groups from same source? | | W | W | W | W | W | W | Y | W | Y | Y | N | N | N | W | Y | N | Y | N | Y | W | W | N | N | Y | Y | Y | Y | Y | Y | Y | Y | N | Y | W | W | Y | N | W | N | W | Y | W | Y | N |
|  | Selected by sleep severity? | | N | N | N | N | N | N | N | N | N | N | N | N | N | N | N | N | N | N | N | N | N | N | N | N | N | N | N | N | N | N | N | N | N | N | N | N | N | N | N | N | N | N | N | N |
|  | ADHD confirmed? | | Y | Y | Y | Y | Y | Y | Y | Y | Y | Y | Y | Y | Y | Y | Y | Y | Y | Y | Y | Y | Y | Y | Y | Y | Y | Y | Y | Y | Y | Y | Y | Y | Y | Y | Y | Y | Y | Y | Y | Y | Y | Y | Y | N |
|  | TD healthy? | | Y | W | Y | Y | Y | Y | Y | W | Y | Y | Y | Y | Y | Y | W | Y | Y | Y | Y | W | Y | Y | Y | Y | Y | Y | Y | Y | Y | Y | Y | Y | Y | W | W | Y | Y | W | Y | W | Y | Y | W | Y |
| Judgment | | | low | moderate | low | low | low | low | low | moderate | low | low | low | low | low | low | low | low | low | low | low | moderate | low | low | low | low | low | low | low | low | low | low | low | low | low | moderate | moderate | low | low | moderate | low | moderate | low | low | low | moderate |
| **Classification of interventions** | Standardized diagnosis tools? | | Y | Y | Y | Y | Y | Y | Y | Y | Y | Y | Y | Y | Y | Y | Y | Y | Y | Y | Y | Y | Y | Y | Y | Y | Y | Y | Y | Y | Y | Y | Y | Y | Y | Y | Y | Y | Y | Y | Y | Y | Y | Y | Y | Y |
|  | Certified diagnosticians? | | Y | Y | Y | Y | Y | Y | Y | Y | Y | Y | Y | Y | Y | N | Y | Y | N | Y | Y | N | Y | N | N | Y | Y | Y | Y | Y | Y | Y | Y | Y | N | N | N | N | Y | N | N | N | Y | N | Y | Y |
| Judgment | | | low | low | low | low | low | low | low | low | low | low | low | low | low | moderate | low | low | moderate | low | low | moderate | low | moderate | moderate | low | low | low | low | low | low | low | low | low | moderate | moderate | moderate | moderate | low | moderate | moderate | moderate | low | moderate | low | low |
| **Deviations from intended interventions** | ADHD treatment ongoing? | | N | N | N | N | N | W | N | Y | N | N | N | N | N | N | N | Y | Y | N | N | N | N | N | N | N | N | N | N | N | N | N | N | N | N | W | W | N | N | N | N | N | N | N | N | Y |
|  | Meds affected sleep? | | - | - | - | - | - | - | - | N | - | - | - | - | - | - | - | W | W | - | - | - | - | - | - | - | - | - | - | - | - | - | - | - | - | - | - | - | - | - | - | - | - | - | - | N |
| Judgment | | | low | low | low | low | low | serious | low | moderate | low | low | low | low | low | low | low | serious | serious | low | low | low | low | low | low | low | low | low | low | low | low | low | low | low | low | serious | serious | low | low | low | low | low | low | low | low | moderate |
| **Missing data** | (Nearly) all participants' data shown? | | Y | W | Y | N | N | Y | Y | Y | Y | Y | Y | Y | Y | W | W | N | W | Y | Y | Y | Y | N | W | N | N | N | N | N | N | N | N | Y | Y | Y | Y | N | Y | Y | N | W | Y | Y | Y | Y |
|  | Similar data missing reasons? | | - | - | - | N | N | - | - | - | - | - | - | - | - | - | - | N | - | - | - | - | - | N | - | Y | Y | Y | Y | Y | Y | Y | Y | - | - | - | - | Y | - | - | N | - | - | - | - | - |
|  | No missing-data bias? | | - | - | - | N | N | - | - | - | - | - | - | - | - | - | - | N | - | - | - | - | - | N | - | N | N | N | N | N | N | N | N | - | - | - | - | N | - | - | N | - | - | - | - | - |
| Judgment | | | low | critical | low | critical | critical | low | low | low | low | low | low | low | low | critical | critical | critical | critical | low | low | low | low | critical | critical | serious | serious | serious | serious | serious | serious | serious | serious | low | low | low | low | serious | low | low | critical | critical | low | low | low | low |
| **Measurement of outcomes** | Operator blinded? | | W | W | W | W | W | W | W | N | W | W | W | W | N | N | N | N | N | Y | Y | Y | W | W | W | N | N | N | N | N | N | N | N | W | Y | W | W | N | W | N | N | W | W | W | Y | N |
|  | Consistent tools? | | Y | Y | Y | Y | Y | Y | Y | Y | Y | Y | Y | Y | Y | Y | Y | Y | Y | Y | Y | Y | Y | Y | Y | Y | Y | Y | Y | Y | Y | Y | Y | Y | Y | Y | Y | Y | Y | Y | Y | Y | Y | Y | Y | Y |
| Judgment | | | moderate | moderate | moderate | moderate | moderate | moderate | moderate | serious | moderate | moderate | moderate | moderate | serious | serious | serious | serious | serious | low | low | low | moderate | moderate | moderate | serious | serious | serious | serious | serious | serious | serious | serious | moderate | low | moderate | moderate | serious | moderate | serious | serious | moderate | moderate | moderate | low | serious |
| **Selection of the reported result** | Pre-registered? | | N | N | N | N | N | N | N | Y | N | Y | N | N | N | N | N | N | N | N | N | N | N | N | N | N | N | N | N | N | N | N | N | N | N | N | N | N | N | N | N | N | N | N | N | N |
|  | All parameters reported? | | Y | Y | Y | Y | Y | W | W | Y | Y | W | W | W | Y | Y | W | Y | Y | W | W | W | Y | Y | W | Y | Y | Y | Y | Y | Y | Y | Y | W | Y | Y | Y | Y | W | W | Y | Y | W | Y | Y | Y |
|  | Subgroups analyzed? | | N | N | Y | N | N | N | N | N | Y | N | N | N | N | N | N | N | N | N | N | N | N | N | N | N | N | N | N | N | N | N | N | N | N | N | N | N | N | N | Y | N | N | N | N | N |
| Judgment | | | serious | serious | moderate | serious | serious | critical | critical | moderate | moderate | serious | critical | critical | serious | serious | critical | serious | serious | critical | critical | critical | serious | serious | critical | serious | serious | serious | serious | serious | serious | serious | serious | critical | serious | serious | serious | serious | critical | critical | moderate | serious | critical | serious | serious | serious |
| **Overall Risk of Bias** | | | **low** | **moderate** | **low** | **moderate** | **moderate** | **low** | **low** | **moderate** | **low** | **low** | **low** | **low** | **low** | **moderate** | **moderate** | **moderate** | **moderate** | **low** | **low** | **low** | **low** | **moderate** | **moderate** | **moderate** | **moderate** | **moderate** | **moderate** | **moderate** | **moderate** | **moderate** | **moderate** | **low** | **low** | **moderate** | **moderate** | **moderate** | **low** | **moderate** | **moderate** | **moderate** | **low** | **low** | **low** | **moderate** |

*Note.*

1. This framework was adapted from the Cochrane ROBINS-I tool with study-specific modifications for sleep research in ADHD.
2. Each item was rated as: Yes (Y), No (N), or No Information (W).
3. Each domain was judged as: Low, Moderate, Serious, or Critical risk of bias.

**Appendix 3 GRADE Evidence Quality Assessment**

| Domain | | Rating criteria | |
| --- | --- | --- | --- |
| Lower if | Risk of Bias | ROBINS-I assessment | Low/Moderate → No change |
|  |  |  | Serious → -1 |
|  |  |  | Critical → -2 |
|  | Inconsistency | Heterogeneity (*I²*) | Low → No change |
|  |  |  | Moderate → -1 |
|  |  |  | High → -2 |
|  | Indirectness | Non-target population included? | → -0.5 |
|  |  | ADHD diagnostic criteria inconsistent? | → -0.5 |
|  |  | TD group health unconfirmed? | → -0.5 |
|  |  | Assessment type of sleep metrics inconsistent? | → -0.5 |
|  | Imprecision | 95% CI includes 0 | → -1 |
|  | Publication bias | Funnel Plot/Egger’s test/Trim-and-Fill | 1 tests positive → -1 |
|  |  |  | ≥2 tests positive → -2 |
| Higher if | Large effect | Effect Size (Hedges’ g) | SMD > 0.5 → +1 |
|  |  |  | SMD > 0.8 → +2 |
|  | Dose response | ADHD severity-sleep correlation analyzed | → +1 |
|  | All plausible residual confounding | Robust to sensitivity tests | → +1 |
|  |  | All key confounders controled | → +1 |

*Note.* This framework was adapted from the GRADE rating tool with study-specific modifications for sleep research in ADHD.

**Appendix 4 Demographic Background Information of Participants across Studies for Meta-Analysis**

| Study | Total | |  | TD | | |  | ADHD group | | | | | | | | |
| --- | --- | --- | --- | --- | --- | --- | --- | --- | --- | --- | --- | --- | --- | --- | --- | --- |
|  | sample size | mean age |  | sample size | mean age | male rate |  | sample size | mean age | male rate | Ittentive Type % | Hyperactive-Impulsive Type % | Combined Type % | medication-naïve % | medication-withdrawn % | currently medicated % |
| Jessica R. Lunsford-Avery,2025 | 56 | 15.275 |  | 29 | 15.420 | - |  | 27 | 15.120 | - | - | - | - | - | - | - |
| Blanka Vojnits, 2024 | 48 | 16.444 |  | 29 | 16.535 | 0.655 |  | 19 | 16.304 | 0.632 | 0.684 | 0.053 | 0.211 | - | - | - |
| Pınar Özbudak,2024 | 67 | 11.057 |  | 32 | 12.770 | 0.281 |  | 35 | 9.490 | 0.829 | 0.629 | 0.114 | 0.257 | - | - | - |
| Tamara Speth,2023 exp1 | 36 | 8.822 |  | 18 | 8.672 | - |  | 18 | 8.973 | - | - | - | - | - | - | - |
| Tamara Speth,2023 exp2 | 30 | 8.850 |  | 15 | 8.700 | 0.800 |  | 15 | 9.000 | 0.800 | - | - | - | - | - | 1.000 |
| Nato Darchia,2022 | 18 | 12.235 |  | 9 | 12.080 | 0.556 |  | 9 | 12.390 | 0.778 | - | - | - | - | - | - |
| Anna Castelnovo,2022 | 53 | 10.337 |  | 23 | 10.150 | - |  | 30 | 10.480 | - | 0.200 | 0.067 | 0.733 | - | - | - |
| Mirjam Ziegler,2021 | 57 | 9.382 |  | 33 | 9.180 | 0.667 |  | 24 | 9.660 | 0.833 | - | - | 0.833 | - | - | - |
| María Fernanda Zerón‑Rugerio,2021 | 120 | 9.300 |  | 60 | 9.300 | 0.567 |  | 60 | 9.300 | 0.567 | 0.367 | - | 0.633 | - | - | - |
| Alaa El-Din M. Darwesh,2021 | 84 | 7.685 |  | 42 | 7.450 | 0.690 |  | 42 | 7.920 | 0.690 | 0.238 | 0.214 | 0.548 | 1.000 | - | - |
| Nato Darchia,2021 exp1 | 18 | 12.235 |  | 9 | 12.080 | 0.556 |  | 9 | 12.390 | 0.778 | - | - | - | - | - | - |
| Nato Darchia,2021 exp2 | 18 | 12.235 |  | 9 | 12.080 | 0.556 |  | 9 | 12.390 | 0.778 | - | - | - | - | - | - |
| Vanessa K. Thoma,2020 | 122 | 12.950 |  | 61 | 12.970 | 0.377 |  | 61 | 12.930 | 0.377 | - | - | - | - | 0.770 | - |
| Federica De Dea,2019 | 17 | 8.528 |  | 9 | 8.330 | - |  | 8 | 8.750 | - | - | - | - | - | - | - |
| Yoshihiko Saito,2019 | 29 | 9.297 |  | 18 | 9.600 | 0.500 |  | 11 | 8.800 | 1.000 | - | - | - | - | - | - |
| Frances Le Cornu Knight,2019 | 32 | 8.776 |  | 18 | 8.960 | 0.500 |  | 14 | 8.540 | 0.786 | 0.071 | 0.143 | 0.786 | - | - | 0.143 |
| Stephen P. Becker,2019 | 302 | 13.175 |  | 140 | 13.180 | 0.443 |  | 162 | 13.170 | 0.648 | 0.741 | - | 0.259 | - | - | - |
| Roumen Kirov,2017 | 88 | 11.300 |  | 22 | 11.420 | 0.864 |  | 24 | 11.190 | 0.833 | - | - | 1.000 | - | - | - |
| Amanda Cremone,2017 | 29 | 6.749 |  | 15 | 6.730 | 0.733 |  | 14 | 6.770 | 0.714 | - | - | - | - | - | - |
| Jared M. Saletin,2017 | 21 | 11.767 |  | 14 | 11.700 | 0.857 |  | 7 | 11.900 | 0.714 | - | - | - | - | - | - |
| Gulcin Akinci,2015 | 43 | 10.000 |  | 15 | 10.000 | 0.600 |  | 28 | 10.000 | 0.714 | 0.250 | - | - | 1.000 | - | - |
| Alexander Prehn-Kristensen,2013 | 32 | 10.850 |  | 16 | 11.100 | - |  | 16 | 10.600 | - | - | - | - | - | - | - |
| Maya Ringli,2013 | 15 | 11.780 |  | 6 | 11.600 | - |  | 9 | 11.900 | 0.889 | - | - | - | - | - | - |
| Nicola Scott,2013 exp1 | 7737 | 0.500 |  | 7573 | 0.500 | - |  | 164 | 0.500 | - | - | - | - | - | - | - |
| Nicola Scott,2013 exp2 | 7587 | 1.500 |  | 7431 | 1.500 | - |  | 156 | 1.500 | - | - | - | - | - | - | - |
| Nicola Scott,2013 exp3 | 7129 | 2.500 |  | 6986 | 2.500 | - |  | 143 | 2.500 | - | - | - | - | - | - | - |
| Nicola Scott,2013 exp4 | 7290 | 3.500 |  | 7143 | 3.500 | - |  | 147 | 3.500 | - | - | - | - | - | - | - |
| Nicola Scott,2013 exp5 | 7202 | 5.750 |  | 7056 | 5.750 | - |  | 146 | 5.750 | - | - | - | - | - | - | - |
| Nicola Scott,2013 exp6 | 7191 | 6.750 |  | 7048 | 6.750 | - |  | 143 | 6.750 | - | - | - | - | - | - | - |
| Nicola Scott,2013 exp7 | 6878 | 9.580 |  | 6741 | 9.580 | - |  | 137 | 9.580 | - | - | - | - | - | - | - |
| Nicola Scott,2013 exp8 | 6290 | 11.670 |  | 6164 | 11.670 | - |  | 126 | 11.670 | - | - | - | - | - | - | - |
| Roumen Kirov,2012 | 39 | 11.250 |  | 19 | 11.260 | 0.895 |  | 20 | 11.240 | 0.950 | - | - | 1.000 | 0.450 | 0.550 | - |
| Reut Gruber,2012 | 75 | 8.610 |  | 49 | 8.690 | 0.612 |  | 26 | 8.460 | 0.654 | 0.308 | 0.038 | 0.654 | - | 0.346 | - |
| Sabrina Wieee,2012 exp1 | 66 | 8.852 |  | 46 | 8.700 | 0.609 |  | 20 | 9.200 | 0.650 | 0.650 | 0.150 | 0.200 | - | 0.200 | - |
| Sabrina Wieee,2012 exp2 | 66 | 8.852 |  | 46 | 8.700 | 0.609 |  | 20 | 9.200 | 0.650 | 0.650 | 0.150 | 0.200 | - | 0.200 | - |
| Alexander Prehn-Kristensen,2011 | 32 | 10.800 |  | 16 | 11.000 | - |  | 16 | 10.600 | - | - | - | - | - | - | - |
| Jiae Choi,2010 | 53 | 8.671 |  | 26 | 8.360 | 0.885 |  | 27 | 8.970 | 0.889 | 0.259 | 0.074 | 0.667 | 1.000 | - | - |
| Reut Gruber,2009 | 38 | 8.736 |  | 23 | 8.610 | 0.565 |  | 15 | 8.930 | 0.667 | 0.133 | 0.067 | 0.800 | 0.533 | 0.467 | - |
| Judith Owens,2009 | 153 | 10.236 |  | 45 | 10.300 | - |  | 80 | 10.200 | - | 0.313 | - | 0.663 | 1.000 | - | - |
| Silvia Miano,2006 | 40 | 8.850 |  | 20 | 8.400 | 0.550 |  | 20 | 9.300 | 0.900 | 0.100 | - | 0.900 | 1.000 | - | - |
| Roumen Kirov,2004 | 34 | 11.200 |  | 17 | 11.200 | 1.000 |  | 17 | 11.200 | 1.000 | - | - | - | 0.176 | 1.000 | - |
| Natali Golan,2004 | 66 | 12.206 |  | 32 | 12.000 | 0.656 |  | 34 | 12.400 | 0.765 | - | - | - | - | 1.000 | - |
| Michel Lecendreux,2000 | 52 | 8.054 |  | 22 | 8.400 | 1.000 |  | 30 | 7.800 | 1.000 | - | - | - | 1.000 | - | - |
| Penny Corkum,1999 | 172 | 9.179 |  | 36 | 9.400 | 0.778 |  | 101 | 9.100 | 0.832 | 0.307 | 0.208 | 0.485 | 0.782 | 0.218 | 0.347 |

**Appendix 5 Primary Sleep Metrics, Assessment Type, and Key Outcomes across Studies for Meta-Analysis**

| Study | Main sleep metrics | Assessment types | Key outcomes (ADHD-TD) |
| --- | --- | --- | --- |
| Jessica R. Lunsford-Avery,2025 | total sleep time | polysomnography | -9.570 |
|  | sleep efficiency |  | -0.010 |
|  | sleep latency |  | 16.380 |
|  | wake after sleep onset |  | 4.320 |
| Tamara Speth,2023 | total sleep time | polysomnography | -14.240 |
|  | sleep efficiency |  | -0.003 |
|  | sleep latency |  | -5.620 |
|  | wake after sleep onset |  | 11.780 * |
| Nato Darchia,2022 | total sleep time | polysomnography | -42.000 |
|  | sleep efficiency |  | -0.019 |
|  | sleep latency |  | 4.800 |
|  | wake after sleep onset |  | 2.900 |
|  | awakening index |  | 0.700 |
| Anna Castelnovo,2022 | total sleep time | polysomnography | -21.730 |
|  | sleep efficiency |  | -0.026 |
|  | sleep latency |  | 6.640 * |
|  | wake after sleep onset |  | 3.870 |
|  | awakening index |  | 0.740 |
| Mirjam Ziegler,2021 | total sleep time | actigraphy | -26.000 |
|  | sleep efficiency |  | -0.045 * |
|  | sleep latency |  | 17.500 *** |
| María Fernanda Zerón‑Rugerio,2021 | total sleep time | actigraphy | -1.600 |
|  | sleep efficiency |  | 0.003 |
|  | wake after sleep onset |  | 2.013 |
| Alaa El-Din M. Darwesh,2021 | total sleep time | polysomnography | -32.620 *** |
|  | sleep efficiency |  | -0.064 *** |
|  | sleep latency |  | 11.700 *** |
|  | wake after sleep onset |  | 17.200 *** |
|  | awakening index |  | 7.180 * |
| Nato Darchia,2021 exp1 | total sleep time | actigraphy | -67.400 |
|  | sleep efficiency |  | -0.023 |
|  | sleep latency |  | 7.200 |
|  | wake after sleep onset |  | 2.700 |
|  | awakening index |  | 0.300 |
|  | stage shift |  | 0.100 |
| Nato Darchia,2021 exp2 | total sleep time | actigraphy | -5.000 |
|  | sleep efficiency |  | -0.010 |
|  | sleep latency |  | 2.200 |
|  | wake after sleep onset |  | 0.200 |
|  | awakening index |  | 0.800 |
|  | stage shift |  | 2.200 |
| Vanessa K. Thoma,2020 | total sleep time | self-report | -11.200 |
|  | sleep latency |  | 10.100 * |
| Frances Le Cornu Knight,2019 | total sleep time | actigraphy | 10.434 *** |
|  | sleep efficiency |  | -0.037 |
|  | sleep latency |  | 19.917 * |
| Stephen P. Becker,2019 | sleep efficiency | actigraphy | -0.006 |
|  | wake after sleep onset |  | 0.430 |
| Roumen Kirov,2017 | total sleep time | polysomnography | 30.890 |
|  | sleep efficiency |  | 0.000 |
|  | sleep latency |  | 2.880 |
|  | wake after sleep onset |  | 2.560 |
| Amanda Cremone,2017 | total sleep time | polysomnography | 2.090 |
|  | sleep efficiency |  | 0.026 |
|  | sleep latency |  | 3.600 |
|  | wake after sleep onset |  | -7.860 |
| Jared M. Saletin,2017 | total sleep time | polysomnography | -20.000 |
|  | sleep efficiency |  | -0.008 |
|  | sleep latency |  | 1.000 |
|  | wake after sleep onset |  | 6.000 |
| Alexander Prehn-Kristensen,2013 | total sleep time | polysomnography | 21.000 |
|  | sleep efficiency |  | -0.017 |
|  | sleep latency |  | 3.400 |
|  | wake after sleep onset |  | 3.000 |
|  | stage shift |  | 0.500 |
| Maya Ringli,2013 | total sleep time | electroencephalography | 19.300 |
|  | sleep efficiency |  | 0.014 |
|  | sleep latency |  | -6.300 |
|  | wake after sleep onset |  | -5.000 |
| Nicola Scott,2013 exp1 | total sleep time | parent-report | -13.000 * |
| Nicola Scott,2013 exp2 | total sleep time | parent-report | -3.000 |
| Nicola Scott,2013 exp3 | total sleep time | parent-report | -6.000 |
| Nicola Scott,2013 exp4 | total sleep time | parent-report | -3.000 |
| Nicola Scott,2013 exp5 | total sleep time | parent-report | -17.000 *** |
| Nicola Scott,2013 exp6 | total sleep time | parent-report | -18.000 *** |
| Nicola Scott,2013 exp7 | total sleep time | parent-report | -14.000 *** |
| Nicola Scott,2013 exp8 | total sleep time | parent-report | -5.000 |
| Roumen Kirov,2012 | total sleep time | polysomnography | 41.140 * |
|  | sleep efficiency |  | 0.007 |
|  | sleep latency |  | -3.330 |
| Reut Gruber,2012 | total sleep time | polysomnography | 0.360 |
|  | sleep efficiency |  | -0.020 |
|  | sleep latency |  | 6.910 |
| Sabrina Wieee,2012 exp1 | total sleep time | polysomnography | 6.400 |
|  | sleep efficiency |  | -0.002 |
|  | sleep latency |  | 4.400 |
| Sabrina Wieee,2012 exp2 | total sleep time | actigraphy | -9.500 |
|  | sleep latency |  | 6.800 |
| Alexander Prehn-Kristensen,2011 | total sleep time | polysomnography | 22.000 |
|  | sleep efficiency |  | 0.001 |
|  | sleep latency |  | -0.100 |
|  | stage shift |  | 0.100 |
| Jiae Choi,2010 | total sleep time | polysomnography | 18.040 |
|  | sleep efficiency |  | 0.015 |
|  | sleep latency |  | -1.070 |
| Reut Gruber,2009 | total sleep time | polysomnography | -33.250 * |
|  | sleep efficiency |  | -0.020 |
|  | sleep latency |  | -1.610 |
| Judith Owens,2009 | total sleep time | actigraphy | -29.090 *** |
|  | sleep efficiency |  | -0.020 * |
|  | sleep latency |  | -1.500 |
| Silvia Miano,2006 | total sleep time | polysomnography | -83.800 *** |
|  | sleep efficiency |  | -0.055 |
|  | sleep latency |  | 7.500 |
|  | wake after sleep onset |  | 2.200 |
|  | stage shift |  | 2.200 * |
| Roumen Kirov,2004 | total sleep time | polysomnography | 36.700 |
|  | sleep efficiency |  | 0.000 |
|  | wake after sleep onset |  | 5.300 |
|  | stage shift |  | 0.300 |
| Natali Golan,2004 | total sleep time | polysomnography | 24.000 |
|  | sleep efficiency |  | 0.017 |
|  | sleep latency |  | -5.000 |
|  | awakening index |  | 3.500 * |
| Michel Lecendreux,2000 | total sleep time | polysomnography | 3.270 |
|  | sleep efficiency |  | -0.002 |
|  | sleep latency |  | 1.760 |

*Note.* The reported Key outcomes represent the mean differences (ADHD group minus TD group). * *p* < 0.05, ** *p* < 0.01, *** *p* < 0.001

**Appendix 6 Overview of Heterogeneity Data for Sleep Metrics in Meta-Analysis**

| sleep metrics | Cochrane Q test | *I²* | Heterogeneity |
| --- | --- | --- | --- |
| Total sleep time | 138.105 *** | 87.5% | high |
| Sleep efficiency | 66.806 *** | 62.6% | moderate |
| Sleep latency | 56.073 *** | 54.6% | moderate |
| Wake after sleep onset | 33.881 ** | 56.4% | moderate |
| Awakening index | 22.676 *** | 73.7% | moderate |
| Stage shift | 7.865 | 36.4% | low |

*Note.* ** *p* < 0.01, *** *p* < 0.001.

**Appendix 7 Results of the meta-regression with assessment type as a covariate**

| sleep metrics | Cochrane Q test | R² | I² adjusted for assessment type | Heterogeneity explained by assessment type |
| --- | --- | --- | --- | --- |
| Total sleep time | 1.186 | 0.0% | 89.5% | none |
| Sleep efficiency | 0.702 | 0.0% | 64.1% | none |
| Sleep latency | 2.872 | 0.0% | 56.5% | none |
| Wake after sleep onset | 2.741 | 16.9% | 49.9% | low |
| Awakening index | 0.254 | 0.0% | 78.3% | none |
| Stage shift | 0.162 | 0.00% | 48.10% | none |

*Note.*

1. All Cochrane Q-test results were non-significant, indicating no statistically significant differences in meta-analyses outcomes across different assessment types.
2. R²= 0 means that the covariate assessment type had no explanatory power for the variance in effect sizes. R²< 20% means that assessment type had low explanatory power for the variance in effect sizes (Viechtbauer, 2010).
3. Except for wake after sleep onset, the heterogeneity of all other sleep metrics did not decrease after including the covariate.

**Appendix 8 Multiple Moderation Model Diagram for Stage Shift**


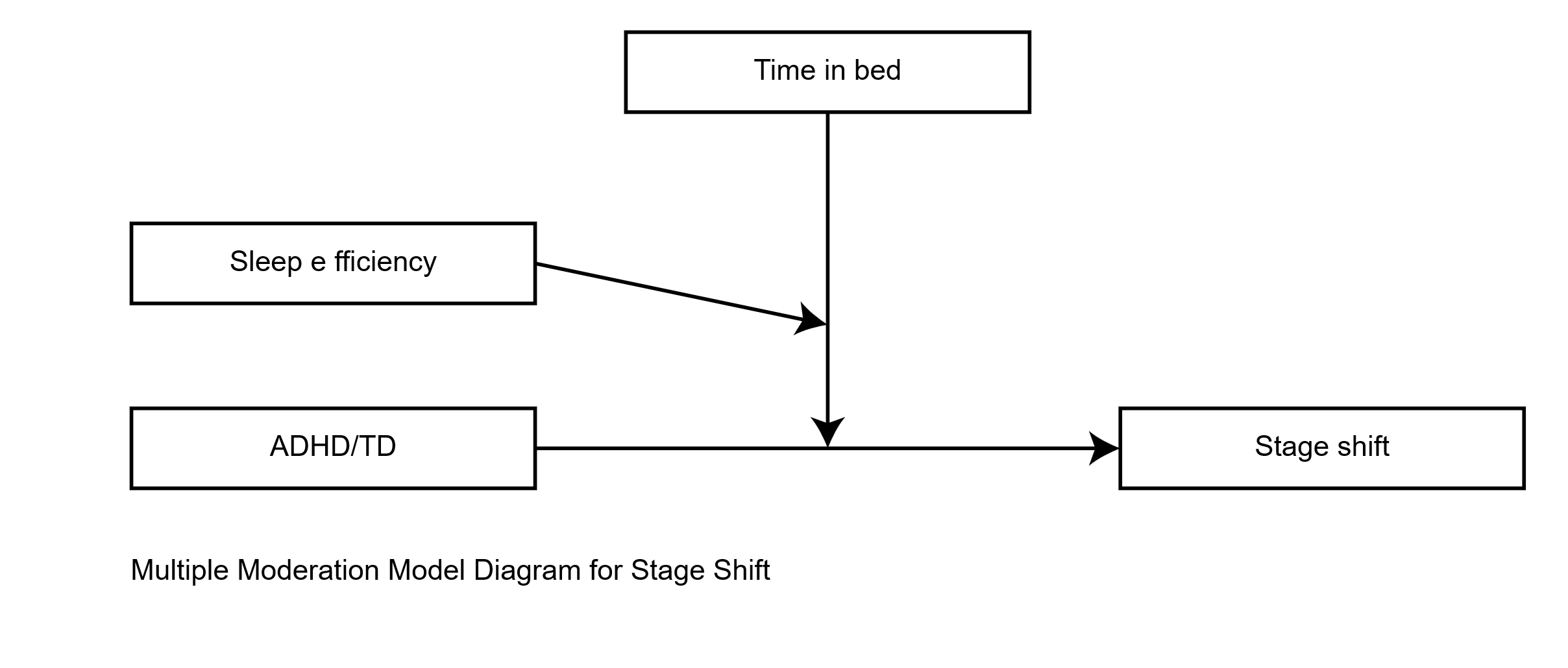


**Appendix 9 Mediation Model Diagram for Awakening Index**


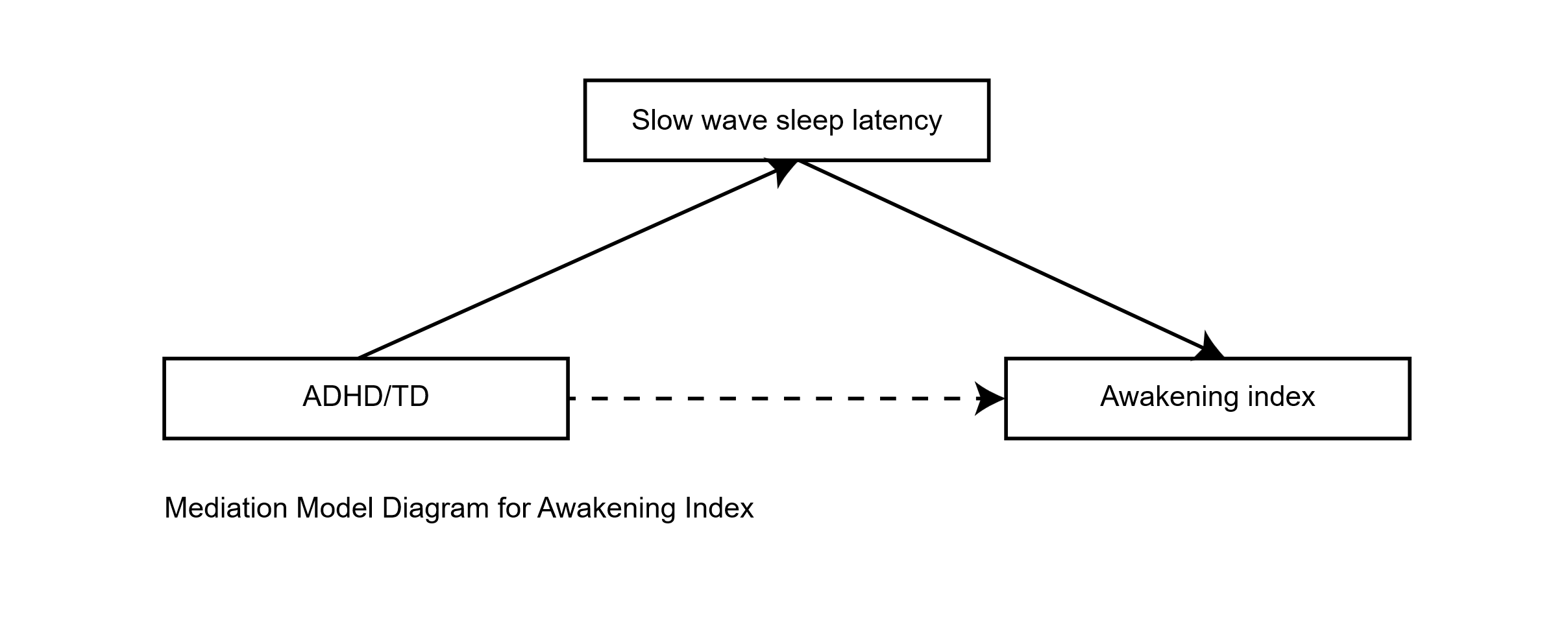


**Appendix 10 Contour-Enhanced Funnel Plots of Included Studies’ Effect Sizes**


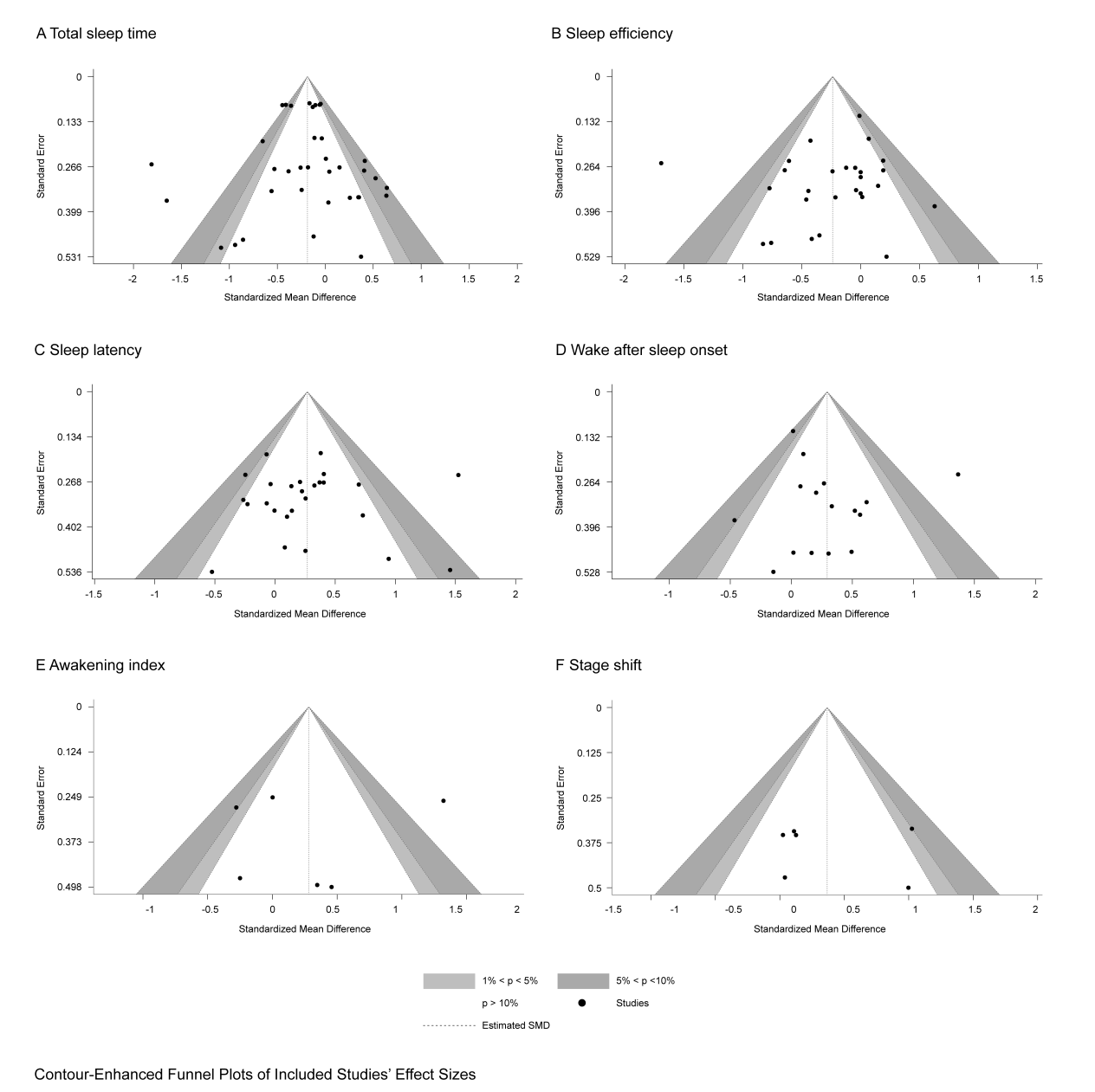


**Appendix 11 Funnel Plot of Imputed and Included Studies’ Effect Sizes Investigating Wake after Sleep Onset and Awakening Index**


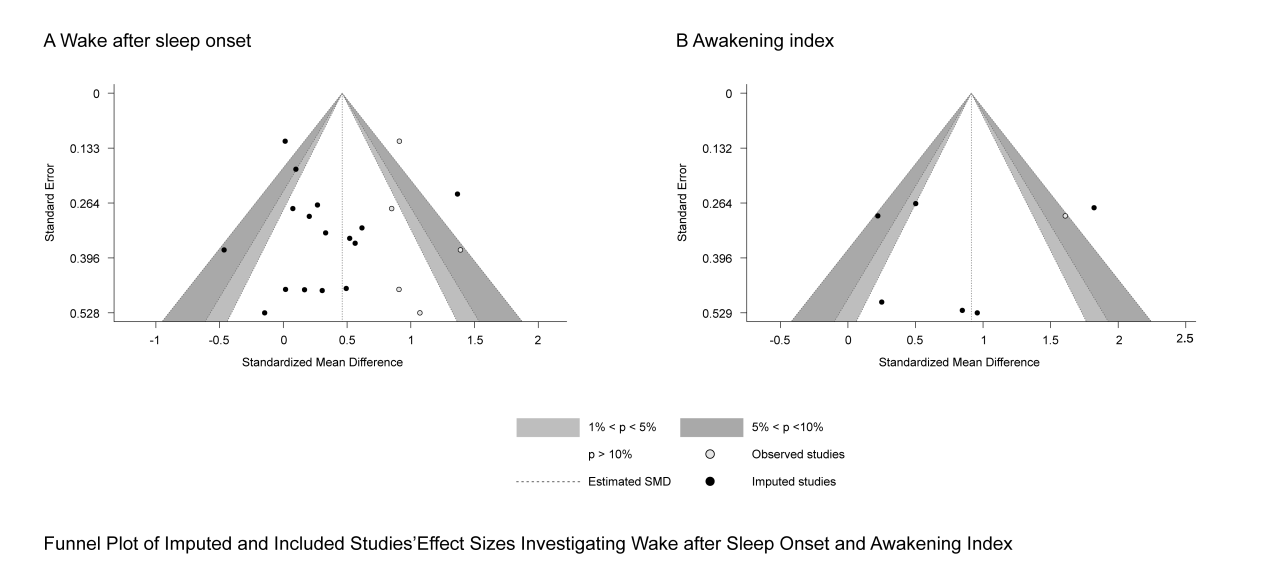


**Appendix 12 Overview of Publication Bias Evaluation Results**

|  | Funnel Plot |  | Trim-and-Fill | | |  | Egger’s test |
| --- | --- | --- | --- | --- | --- | --- | --- |
|  | symmetry |  | Abnormal study | Adjusted | |  | z |
|  |  |  |  | SMD | 95%CI |  |  |
| Total sleep time | Yes |  | 0 | - | - |  | -0.107 |
| Sleep efficiency | Yes |  | 0 | - | - |  | -0.319 |
| Sleep latency | Yes |  | 0 | - | - |  | 0.189 |
| Wake after sleep onset | Yes |  | 5 | 0.462 | 0.244, 0.679 |  | -0.207 |
| Awakening index | Yes |  | 1 | 0.913 | 0.402, 1.424 |  | -0.287 |
| Stage shift | Yes |  | 0 | - | - |  | 0.320 |

*Note.* All Egger’s test results were non-significant.

**Appendix 13 Rating of Evidence Quality for Sleep Metrics**

| sleep metrics | Lower if | | | | |  | Higher if | | |  | Judgement |
| --- | --- | --- | --- | --- | --- | --- | --- | --- | --- | --- | --- |
|  | Risk of Bias | Inconsistency | Indirectness | Imprecision | Publication bias |  | Large effect | Dose response | All plausible residual confounding |  |  |
| total sleep time | - | -2 | -1 | - | - |  | - | - | - |  | very low |
| sleep efficiency | - | -1 | -1 | - | - |  | - | - | - |  | very low |
| sleep latency | - | -1 | -1 | - | - |  | - | - | - |  | very low |
| wake after sleep onset | - | -1 | -1 | - | -1 |  | - | - | - |  | very low |
| awakening index | - | -1 | - | - | -1 |  | +1 | - | - |  | low |
| stage shift | - | - | - | - | - |  | - | - | - |  | low |
